# Physical, Spatial, and Molecular Aspects of Extracellular Matrix of *In Vivo* Niches and Artificial Scaffolds Relevant to Stem Cells Research

**DOI:** 10.1155/2015/167025

**Published:** 2015-08-16

**Authors:** Maria Akhmanova, Egor Osidak, Sergey Domogatsky, Sergey Rodin, Anna Domogatskaya

**Affiliations:** ^1^Imtek Limited, 3 Cherepkovskaya 15, Moscow 21552, Russia; ^2^Gamaleya Research Institute of Epidemiology and Microbiology Federal State Budgetary Institution, Ministry of Health of the Russian Federation, Gamalei 18, Moscow 123098, Russia; ^3^Russian Cardiology Research and Production Center Federal State Budgetary Institution, Ministry of Health of the Russian Federation, 3 Cherepkovskaya 15, Moscow 21552, Russia; ^4^Division of Matrix Biology, Department of Medical Biochemistry and Biophysics, Karolinska Institute, 171 77 Stockholm, Sweden

## Abstract

Extracellular matrix can influence stem cell choices, such as self-renewal, quiescence, migration, proliferation, phenotype maintenance, differentiation, or apoptosis. Three aspects of extracellular matrix were extensively studied during the last decade: physical properties, spatial presentation of adhesive epitopes, and molecular complexity. Over 15 different parameters have been shown to influence stem cell choices. Physical aspects include stiffness (or elasticity), viscoelasticity, pore size, porosity, amplitude and frequency of static and dynamic deformations applied to the matrix. Spatial aspects include scaffold dimensionality (2D or 3D) and thickness; cell polarity; area, shape, and microscale topography of cell adhesion surface; epitope concentration, epitope clustering characteristics (number of epitopes per cluster, spacing between epitopes within cluster, spacing between separate clusters, cluster patterns, and level of disorder in epitope arrangement), and nanotopography. Biochemical characteristics of natural extracellular matrix molecules regard diversity and structural complexity of matrix molecules, affinity and specificity of epitope interaction with cell receptors, role of non-affinity domains, complexity of supramolecular organization, and co-signaling by growth factors or matrix epitopes. Synergy between several matrix aspects enables stem cells to retain their function in vivo and may be a key to generation of long-term, robust, and effective in vitro stem cell culture systems.

## 1. Introduction

Stem cells are a major focus in regenerative medicine, since they promise to provide unlimited amounts of cells for transplantation. Stem cells within their natural niches* in vivo* maintain through the lifetime and retain ability to serve the regenerative purposes by making choices for survival, self-renewal, differentiation, quiescence, or apoptosis in regulated manner. It would be a breakthrough achievement to learn how to maintain the functional versatility of stem cells cultured through years in* ex vivo* culture. Thus, stem cell differentiation could be manipulated* in vitro *in efficient and safe way.

Stem cell behavior patterns are guided by the external signals that a stem cell receives from its local niche. Such cues include soluble growth factors and hormones, contacts from neighboring cells, and also cues from local extracellular matrix (ECM) [1, 2]. All these factors act in concert* in vivo*, so that stem cells maintain their state and make proper regenerative choices through the lifetime.

ECM was considered to be an inert supportive scaffold for the cells just 40 years ago. Fibronectin, laminin (laminin-111), and several other ECM molecules have been used in cell culture since they made culturing convenient and also improved cell survival [3, 4]. The support provided by ECM to the cultured cells has been assessed mainly in terms of cell survival and high adhesiveness to the matrix and/or high degree of cell spreading. Steven Frisch and Hunter Francis in 1994 introduced term “anoikis” which described cell apoptosis caused by “homelessness,” lack of biologically relevant ECM adhesion contacts [5]. Anoikis is a natural mechanism that allows keeping cells restricted to their natural niches and self-destructing the cells that drifted away from the natural niches in uncontrolled way. It has been also discovered that several different laminin isoforms are niche-restricted and demonstrated that the adhesive ECM molecules may drastically differ in their effect on the cells (reviewed in [6, 7]). The contact with non-relevant ECM ligand, even highly adhesive, would sometimes result in activation of non-relevant intracellular signaling pathways and alteration of behavioral patterns. For instance, it could be induction of apoptosis, loss of phenotype, or malignant transformation [8, 9]. It became evident that biological relevance of external cues that the cell receives is essential for long-term functionality and maintenance of the cell.

**Figure 1:**
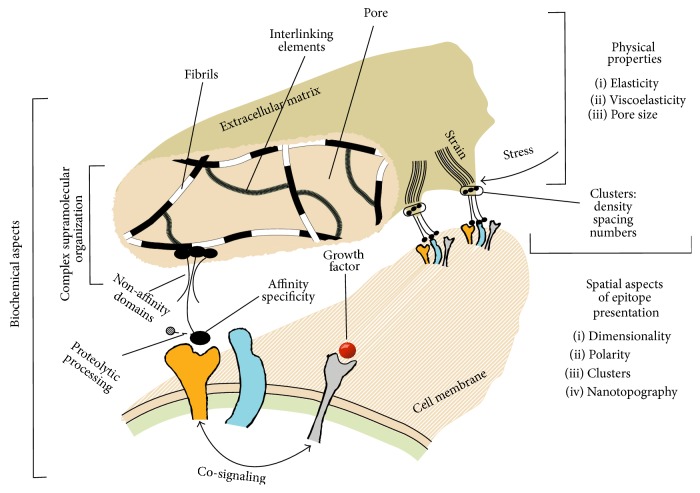
*Physical, spatial, and molecular aspects of extracellular matrix that are known to affect stem cell behavioral patterns and choices*. Extracellular matrix (ECM) of mammalian tissues* in vivo* is a complex structure composed of multiple molecular components, such as fibrils, fibril-associated crosslinking elements, and specific ligands interacting with cell receptors. Such molecular complexity has a biological reason, since lack of ECM molecules, due to mutation or knockout, often results in pathology or even mortality. Molecular composition of a matrix composition and the way of structural arrangement of the molecular components determine the physical, spatial, and molecular characteristics of the scaffold which, as we demonstrate in the review, may actively affect stem cells behavioral patterns. Physical aspects include stiffness (or elasticity); viscoelasticity; pore size and porosity; amplitude of static and dynamic deformations of the matrix (tensile, compressive, or shear); and frequency of cyclic deformations. Due to complex organization, elastic properties of the natural ECM cannot be characterized by a single parameter of Young's modulus (which is valid for many synthetic gels). The stress-strain relation is often nonlinear and is described by stress-strain curve; the natural ECM tend to rearrange their structure under stress, which makes them viscoelastic and prone to plastic deformation. Viscoelastic materials change their elastic properties when they are subject to static strains or cyclic (dynamic) deformations; therefore, one has to take tensile characteristics of the system into account. Spatial arrangement includes dimensionality (2D or 3D) of the scaffold introduced to the cell; thickness of the substrate layer underlying the cell; cell polarity; surface area and geometry of adhesion surface; microscale topography of the surface; epitope concentration; epitope clustering characteristics (number of epitopes per cluster, spacing between epitopes within cluster, spacing between separate clusters, cluster patterns, and order or disorder in epitope arrangement); size, shape, and level of disorder of nanotopographical features such as fibers diameter and orientation. Molecular properties concern structural complexity of ECM molecules, types of adhesion epitopes and corresponding receptors, co-signaling (cooperation of growth factor- and matrix-dependent receptors), and affinity interactions.

During the last decade, three different areas of knowledge related to biological relevance of the ECM cues to the cells have developed extensively (see Figure 1). Each area presented genuine insights that resulted in efficient, safe, and robust stem cell culture protocols.

The first area of knowledge regards* physical properties* of ECM: stiffness (or elasticity); viscoelasticity; pore size and porosity; amplitude of static and dynamic deformations of the matrix (tensile, compressive, or shear); and frequency of cyclic deformations. Mesenchymal stem cells (MSCs) and other types of stem cells differentiate according to stiffness of surrounding matrix [10, 11]. Viscoelasticity of the matrix affects sensing of stiffness by cells because of creep and stress-relaxation [12]. Tensile, compressive, or shear stresses cause deformation of the matrix that changes its stiffness and provide signals to the cell through cytoskeleton reorganization [13]. Dynamical characteristics of ECM deformations such as strain rate or load frequency are also the factors that can affect stem cell fate [14]. The pathway mechanisms of mechanotransduction are essentially identified with emphasis on myosin role in cell contractility and force-sensing [15].

The second area of knowledge regards* spatial organization* of the adhesion epitopes presented to the cell, which comprises dimensionality; thickness of the substrate layer; cell polarity; size, shape, and topography of adhesion surface; epitope concentration and epitope clustering (characterized by number of epitopes per cluster, spacing between epitopes within cluster, spacing between separate clusters, cluster patterns, and level of disorder in epitope arrangement); and arrangement of nanotopographical obstacles. Difference between two-dimensional (2D) and three-dimensional (3D) matrices in guiding stem cell fate is essential, as well as cell polarity that is defined by placement of epitopes [16]. Size and shape of adhesion surface may govern cell size and shape (morphology) as has been established by island micropatterning method [17]. It is also known that density and distribution of epitopes, such as grouping into clusters, influence cells response. These characteristics of substrate guide integrin attachments and interplay between integrin molecules, which is a controlling step in signal transduction to the cell. Topographical features on the substrate such as grooves or pillars of micrometer to nanometers size are also sensed by cells via arrangement of adhesion epitopes available to the cell [2, 18].

The third area of knowledge regards* biochemical complexity* of natural ECM molecules and supramolecular structures formed by the ECM molecules. The major issues in this area of knowledge regard diversity and structural complexity of matrix molecules; affinity and specificity of epitope interaction with cell receptors; role of non-affinity domains; ability to assemble into complex supramolecular structures due to structural domains of specific shape; and co-signaling enabled by cell interaction with several matrix epitopes or growth factors. ECM molecules, such as laminins and collagens, are large and complex protein molecules, with molecular weight up to one million Daltons. There must be a strong biological reason for such molecular complexity. The role of ECM molecules spreads far beyond mere presentation of the specific adhesion epitope to cell receptors. We discuss concept of* co-signaling*: signaling enabled by collaboration between different types of cell receptors, for instance, by ECM receptors and growth factor receptors. Co-signaling allows achieving synergetic effects that neither of the participating activated cell membrane receptors can cause alone. We discuss whether the affinity epitopes or truncated versions of ECM molecules alone would be as functional as the full-size molecules, the concepts of growth factor accumulation, and presentation to the growth factor receptors [19], as well as the role of a single complex ECM molecule serving as a double-signaling agent [20].

The named three areas have achieved significant progress; however, they have evolved rather independently from one another. It often happens that experimental models that are highly advanced and biologically relevant in one aspect are far from relevant in the other aspects. For instance, experimental models that allow control over physical properties of ECM such as stiffness typically employ small adhesion molecules, for instance, short peptides, instead of natural full-size multidomain matrix molecules. Short peptides immobilized on abnormally stiff surfaces are also a standard approach to study effect of spatial placement of epitopes. Biologically relevant full-size ECM adhesion molecules, such as niche-specific laminin isoforms, fibronectin, or vitronectin, are often immobilized on flat and abnormally stiff plastic or glass surfaces. Conventional 2D-adherent cell cultures for certain cell types result in wrong polarity for growth factors presentation, which may hinder the growth factor signaling.

The review aims to stress the importance of all the three aspects: physical properties, spatial arrangement, and, finally, biochemical complexity of ECM molecules. Over 15 significant characteristics of ECM that may affect the stem cell fate are discussed in the review, supplemented by examples from stem cell research that illustrate significance of the characteristics. The review considers the principle of synergy between different niche factors, which may explain unique ability of stem cell niches* in vivo* to self-renew and attend to regenerative purposes for years with extremely low probability of developing malignancy, suffering total loss of regenerative potential, or making false differentiation choices. Several examples of such synergy between the niche factors are presented hereby, wherein the positive effect for a cell in culture is attained only by combination of two or more relevant niche components. Mimicking natural stem cell niches in all the aspects, physical, spatial, and biochemical, would allow further advances in stem cell technologies and result in efficient, safe, and long-term functional stem cell technologies for regenerative medicine.

## 2. Physical Properties of Extracellular Matrix

Physical characteristics of ECM include a range of microscopic and macroscopic properties, which influence matrix response to the force applied from outside of the tissue and by the cells themselves. The milestone works, such as [10, 21, 22], clearly demonstrated that stem cells can perceive the physical characteristics of surrounding matrix and act accordingly. In the following sections, we provide a short insight into several physical characteristics of the matrix of high biological relevance: stiffness (or elasticity); viscoelasticity; pore size and porosity; amplitude of static and dynamic deformations of the matrix (tensile, compressive, or shear); and frequency of cyclic deformations (see Figure 1 for illustration). We shall demonstrate their ability to influence stem cell fate and discuss possible mechanisms that are involved.

### 2.1. Elasticity

When the material is subjected to tensile (or compressive) force, it will typically elongate (or compress, resp.) in the direction of the applied force [23]. The ratio of the deformation to the total length of the object is termed strain. The load divided by the surface area of the specimen, perpendicular to the load, is termed stress. Material is considered to be elastic, if the relation between stress and strain can be described by the same stress-strain curve, which does not alter after repeated deformations. Ratio between stress and strain is called Young's modulus, often denoted by *E*. It describes the stiffness of a material (also often referred to as rigidity, elasticity, or tissue modulus), that is, its resistance against deformation when subjected to a given stress. Behavior of elastic materials that possess a linear stress-strain curve can be well described with this single parameter.

Biological tissues like most polymer gels exhibit linear elastic behavior at minor deformations, typically within 5% strain [24]. If, however, the stress-strain curve is nonlinear which is typical for many polymeric materials at strains exceeding 5%, it is called nonlinear elasticity, which is the case for tissues* in vivo* [25]. If after high strains the material does not return to its original shape it is called plastic, for example, bone tissue [23].

Tissues usually stiffen under compression due to fluid outflow from the matrix and also stiffen during stretching, but, due to other factors, such as orientation of fibrils parallel to tension vector. For these reasons, stress-strain characteristics of the material can differ significantly for stretching and for compression (e.g., in cartilage tissue [26]).

Shear deformation occurs if deformation is parallel to the surface to which the sheet of tissue or the cell is anchored. For instance, vascular endothelial cells are subject to shear stress in blood vessels due to fluid flow, which is parallel to the blood-facing cell surface. Most tissues in mammalian organisms are subject to shear deformations, as well as certain cell types, for instance, vascular endothelial cells or gut epithelial cells. Shear stress is proportional to shear strain with the coefficient *G* (shear modulus). Shear modulus (*G*) of the material is related to Young's modulus, and for biological materials that are almost incompressible *E* approximately equals three times *G*. The shear modulus *G* is often used to describe material properties of tissues or gels.

#### 2.1.1. Elastic Properties of Mammalian Tissues and Stem Cell Niches

It is difficult to qualify elastic properties of many biological tissues in terms of mere stiffness (Young's modulus), since natural tissues architecture is far more complex compared to synthetic polymers such as polyacrylamide (PAA) gels. Most biological tissues consist of multiple molecular components; for instance, basement membranes contain two intertwining, independently cross-linked networks with very different mechanical properties, collagen IV-based and laminin-based. Certain tissues are multilayered, such as arterial wall, and each layer has its own elastic properties, which enables functional nonlinearity of the stress-strain curve. Most tissues stiffen rapidly with strain. There is viscous component in tissue behavior (see below in Viscoelasticity). Nevertheless, it is possible to measure the elasticity of the “resting tissues” at the beginning of stress-strain curve, where it is close to linear [27]. Note that these values change considerably in case of large strains.

Elasticity of mammalian tissues, characterized by Young's modulus [28–32], ranges from about 0.1 kPa (brain or bone marrow) [11, 33, 34] to about 10 GPa (bone) [35, 36], thus giving range of stiffness levels difference as high as 100,000,000. Values of different biological tissues elasticity are summarized in Table 1. Methods of Young's modulus measurement of biological tissues are shortly reviewed in Table 2.

Stiffness of stem cell niches* in vivo* often is less than mean rigidity of the host tissue. For instance, matrix of chondrons, special zones in cartilage where chondrocytes and stem cells are located, is softer, compared to intercellular areas of cartilage: its Young modulus is 20–30 kPa [37], which is about 30 times less than the hard cartilage tissue [38, 39]. The most rigid matrix that hosts stem cells is precalcified bone with maximum stiffness around 50 kPa [10, 11], that is, over 10,000 times less compared to mature calcified bone. The most soft stem cell niche is probably that of MSCs, which reside in bone marrow. Macroscopic measurements of extracted bone marrow suggest an elasticity of 0.3 kPa [40], as an extracellular space of it is nearly liquid. The central marrow is shown to be generally soft (<0.3 kPa) while surrounding bone is invariably rigid (>1000 kPa) as measured by atomic force microscopy [41].

As the modulus and other material properties emerge from matrix composition and mechanical properties of molecules composing its scaffold, they change with age or disease as in fibrosis. This change is due to alterations in ECM composition, for instance, the lengths and density of the collagen fibrils [23]. Thus, stiffness of fibrotic scar in heart tissue has been shown to exceed Young's modulus of normal tissue by 2 to 3 times and can be from 20 kPa to 60 kPa, as has been measured for fibrotic scars developed after an acute myocardial infarction or after chronic stimuli [42, 43]. Stiffness of fibrotic liver tissue (8–12 kPa) is also 2 to 3 times higher than that of normal liver (2–6 kPa) [44] (Table 1).

#### 2.1.2. Stem Cells and Matrix Elasticity* In Vivo*

Stiffness of the adjacent tissue affects stem cell fate* in vivo* when the cell exits its niche and starts to participate in regenerative process [13]. Stem cells tend to proliferate, migrate towards the injured site, and differentiate to the relevant cell type, adoptive to stiffness of the substrate. Stiffness of ECM has been shown to be crucial for maintenance of satellite stem cells* in vivo* [45]. Collagen VI has been proved to be the major regulator of stiffness in the stem cell niche in this case [45].

Mechanical regulation may cause cell dysfunction if the microenvironment is abnormally rigid. Damaged tissue accumulates excess of ECM components thus becoming too stiff. A rigid scar tissue formed after heart attack permits extremely low rate of MSCs differentiation after cardiac transplantation [46]. It even induces osteogenic differentiation of MSCs injected into the muscle promoting bone formation [13, 47].

#### 2.1.3. Stem Cells and Matrix Elasticity* In Vitro*

To date several hundreds of research papers are dedicated to dependence of stem cell fate on stiffness of their substrates* in vitro.* Most of them consider MSCs. Similar results are obtained with different materials used as substrates: MSCs tend to differentiate to the cell type relevant to stiffness of the substrate as long as the other parameters (such as different substrate geometries or adhesion ligands) are not limiting for cell attachment and spreading [22, 48, 49]. In most experiments elastic materials, such as polyacrylamide (PAA) gels [10], hydrogels of polyethylene glycol (PEG) [50, 51], or hyaluronic acid (HA) [52], are used (for details of matrices see Table 3). MSCs are cultured on substrates of different stiffness in differentiation media specific for the particular cell lineage and expression of specific cell markers is monitored.

Briefly, these and other experiments show that the MSCs differentiate into neuronal or glial cells on the soft matrices that resemble soft brain tissue [10, 53–55]. They differentiate into adipocytes on twofold stiffer substrates [49, 56], into myoblasts on 10-fold stiffer substrates [10, 57], and into osteocytes on harder matrices that mimic premineralized bone [10, 49, 58–60]. Similar results are obtained on stem cells seeded in 3D matrices compared to 2D substrates [61]. MSCs differentiation tendency with respect to substrate stiffness is shown in Table 4.

MSCs have weaker cell adhesion to soft substrates [62] but anchor more strongly to stiff substrates [63]. The level of adhesion strength correlates with commitment of the MSC to specific cell lineage. Suppression of adhesion strength for a cell on hard substrates imitates cell behavior on soft substrates in terms of the lineage marker expression, as shown for MSCs on polyacrylamide (PAA) gels [62]. Alterations in the number, stability, and strength of the developing cell adhesions lead to reorganization of the cytoskeleton and change in cell morphology. It is a crucial step prior to differentiation. On stiffer substrates stem cells tend to spread more and tend to assemble their cytoskeleton, such as build long actin-myosin stress fibers [11]. Majority of MSCs develop branched morphology with multiple filopodia on soft gels that mimic elasticity of brain (0.1–1 kPa) [10]. MSCs acquire rather spheroid shape on matrices resembling adipose tissue (4 kPa) [49]. Spindle-shaped cells appear on stiffer matrices that mimic elasticity of striated muscle (8–17 kPa). Spreading on even more stiff matrices (25–40 kPa) yields polygonal MSCs similar in morphology to osteoblasts [10]. Accordingly, MSCs in stiff 3D matrices of 15 kPa demonstrate a striated morphology and larger cell area than in soft 3D matrices of 2 kPa [61].

Also stiffer hydrogels generally promote acceleration of stem cell proliferation compared to softer gels. It has been established for human bone marrow stem cells on polyvinyl alcohol gels of 1 kPa to 24 kPa stiffness [53] and for rat bone marrow stem cells on polyacrylamide (PAA) substrates of 6.1 kPa and 46.7 kPa [54]. The human MSC proliferation rate increases up to 10-fold with the increase of stiffness from 0.7 kPa to 80 kPa on the polyacrylamide (PAA) substrates [22]. The murine embryonic stem cells (ESC) proliferation accelerates as stiffness increases from 41 kPa to 2.3 MPa on the polydimethylsiloxane (PDMS) substrates [64].

Conclusively, embryonic stem cells (ESC) [64] and adult stem cells of different etiology [65], such as neuronal [21] or adipose cells [66], respond to substrate stiffness in a similar way to MSCs. Hematopoietic stem and progenitor cells* in vitro* adhere better to stiffer substrates and increase migration rate [67, 68]. Also there are several studies investigating MSCs ability to migrate up the stiffness gradient [69, 70]. The following reviews provide further detailed information [11, 13, 15].

#### 2.1.4. Cell Perception Depth of the Matrix Layer

An important question in interpreting experiments described above is whether cells can feel substrate underneath the matrix layer of interest. To address this question, Engler et al. [10] report finding that a thin soft gel on glass (of 0.5–1 *μ*m thickness) fosters cell spreading similar to that of cells on stiffer gels. Also it is known that cells can contract their matrices up to 1–3 *μ*m [71]. It means that, on thin (500 nm) soft gels attached to glass, cells are expected to act as on an effectively stiffer matrix [10].

To understand in detail how deeply cells feel into a matrix, Buxboim et al. [72] have prepared series of thin and elastic polyacrylamide (PAA) gels that are affixed to a rigid substrate. Gels of nominal stiffness of about 1 kPa and varying thickness have been coated with type-I collagen and MSCs have been plated and cultured. By measuring the spread area of cells, which appear to decrease hyperbolically with gel thickness, MSCs have been shown to sense a rigid surface less than 5 *μ*m beneath them, establishing a tactile length scale, with a more modest response seen at 10–20 *μ*m. Notably the thickness of basement membranes* in vivo* (up to 0.3 *μ*m) is significantly lower compared to the thickness perception level determined by the study (from 5 *μ*m), which raises a question whether cells residing on basement membranes* in vivo* get stiffness cues not only from basement membrane itself, but also from underlying tissues.

In most studies, using 2D culture systems to investigate forces and feedbacks of mechanical behavior of the cells, the elasticity-controlled polymer substrates attached to glass or plastic are approximately 50–100 *μ*m thick [10, 72], though, for this thickness, it can be readily assumed that only the polymer substrata influence cells fate. However, not only such quasi-2D systems are appropriate to study cells residing on membrane* in vivo*. Differentiation of MSCs to vascular endothelial cells has been observed even in 3D matrix that exhibits the same elasticity characteristics (2-3 kPa) as intima basement membrane [61].

Studies of matrix stiffness effect on stem cells for different polymer matrices provide similar range of stiffness values for lineage commitment in 2D and 3D, at least for neurogenic, adipogenic, myogenic, and osteogenic differentiation (see Table 4).

#### 2.1.5. Mechanisms of Elasticity Sensing

Cells residing in ECM stabilize their shape by finding balance between tensile and compressive forces. This balance can be achieved passively by cells inner scaffold (cytoskeleton), which is a network of filaments that are either compression-resistant (microtubules) or tension-resistant (microfilaments and intermediate filaments) [12]. Notably, this so-called tensegrity-structure [73] of the cell includes nuclear skeleton [74].

Also, cells can actively apply forces to the matrix and thus deform it. Cells pull the matrix using contracting elements of cytoskeleton (myosins) that are attached to ECM molecules via integrin-mediated connections in the focal adhesion sites. Such pulling is usually termed traction [10]. Cytoskeleton is maintained under prestress to balance the traction stresses. Many cells including MSCs stiffen in response to elevation of substrate stiffness [10, 75].

Cells tend to spread when a stiff matrix does not deform on traction and exhibit round or branched morphology when matrix is soft and deforms easily. The resulting change in MSC morphology determines the fate of the stem cell [12].

It is suggested that cells sense a substrate with high stiffness in a similar way to a substrate with low stiffness which stiffens with strain (reviewed in [1]). As the cell pulls on strain-stiffening substrate, the flexible linkers in the gel rapidly stretch to the maximum length so the cell will perceive it as stiff. Therefore, strain-dependent properties of the matrix are important for the cell behavior [1, 76].

#### 2.1.6. Signaling Pathways Involved in Elasticity Mechanosensing

Cellular active forces can be either isometric or isotonic. They are controlled by the individual systems of protein association, which are partly merged. Isometric tension defines force that pulls on ECM by actomyosin-based contractile mechanisms while maintaining length of the stress fibers. This system is controlled by the Rho A/ROCK pathway. Isotonic tension is caused by actin assembly during stretching of the cell, as it widens spread area. It is exerted via the Rac or Cdc42 pathway on lamellipodia or filopodia [12].

Key elements of the signaling systems that regulate pulling ability of the cell cytoskeleton are the following.

*Integrins*. Stem cells express specific integrins, which connect cytoskeleton to the ECM. The level of cell surface integrins appears to be significantly lower on soft substrates than that on stiff substrates [77]. Different types of integrins are responsible for adhesions at different stiffness levels. Thus, integrin *α*2 is upregulated in the course of osteogenic induction of MSCs on stiff matrices [78]. Integrin *α*5 is downregulated on soft gels, but its overexpression had no effect on cell spreading [15]. Activation of integrin *β*1 in bone marrow MSCs is induced by soft substrate to a significantly greater degree than by stiff substrate [77]. *β*1-integrin signaling in the niche is involved in the maintenance of epidermal stem cells or neural stem/progenitors in a stem cell state [79]. Proliferation of MSC is mediated by activation of integrin *β*1 and selectin [80]. In Section 4 we shall discuss in detail the biochemistry of integrin interaction with the specific matrix molecules, for instance, the laminin family. 

*Myosins*. Ubiquitous component of mechanotransduction is nonmuscle myosin II. It is likely to be involved in generating increasing levels of tension through focal adhesions and thus in mechanisms of sensing matrix elasticity [10]. The very first stages of cell differentiation in embryogenesis are blocked after knockout of force-generating myosins [81]. Inhibition of myosin in MSCs blocks differentiation into all lineages on both rigid and compliant substrates [10]. 

*Rac, RhoA Pathway, and Rho Kinase Effector ROCK*. It is established that signals from growth factor receptors and integrins influence stem cell motility, contractility, and focal adhesions development through Rac and Rho GTPases (which are G proteins). Rac drives motility through actin assembly, and RhoA regulates contraction of actin stress fibers by myosins [11]. Inhibition of the RhoA kinase effector ROCK results in deactivation of myosin. Consequently, ROCK inhibition selectively blocks rigidity-driven osteogenesis but does not affect cell differentiation on compliant substrates. And, on the contrary, RhoA/ROCK activation stimulated osteogenesis but inhibited adipogenesis [82]. RhoA and ROCK may mediate the substrate rigidity-regulated Ca^2+^ oscillation, which determines the physiological functions of human MSCs [83].

### 2.2. Viscoelasticity

Material is called* viscoelastic* if the stress-strain dependence is described not by a single curve but by a hysteresis loop, which is a loop between build-up and release curves. Such material has both elastic and viscous (dissipative) structural elements. When subjected to a constant strain, the viscoelastic material exhibits stress-relaxation: material becomes less stressed with time because of dissipation of elastic energy by its viscous part flow (e.g., fluid movement). When subjected to a constant stress, the viscoelastic material undergoes creep, which is gradual increase in strain with time.

Most biological tissues, including skin, tendon, arterial wall, and cartilage, are viscoelastic materials [23]. One reason is due to the flows of viscous fluids impregnated into the tissue that occur during deformation process. The other cause of viscoelastic properties of tissues is relative sliding of macromolecules of the matrix scaffold [23]. Viscoelasticity of incorporated cells contribute to tissue viscoelasticity in tissues with high cell content such as arterial wall [25].

Viscoelastic materials usually are described by complex shear modulus G, which consists of the elastic component, the storage modulus, and the dissipative or viscous component, the loss modulus. The higher the magnitude of the viscous component of a matrix is, the more the substrate will creep with time under applied force [12]. Values of loss modulus of the skin are in the range of 10 to 300 Pa [84], while for tendon it is in the order of 1 GPa [85].

The creep or the stress-relaxation process takes time; slope of relaxation kinetics curve is characterized by relaxation time. That is why viscoelastic measurements depend on the rate of stress or strain changes and, consequently, on the frequency of the applied dynamic forces.

#### 2.2.1. Stem Cells Traction on Viscoelastic Substrate

Although biological tissues are mostly viscoelastic, only elastic properties of cell substrates have been considered by researches until recently. The pioneering work has been introduced by Cameron et al. [12]. Authors have assumed that as adherent cells begin to exert force on a viscoelastic substrate via their focal adhesions, in contrast to a purely elastic substrate, this substrate will be prone to creep. It may result in cells feeling a time-dependent reduction in the resistive force that they experience when actively pulling the substrate. This reduction in resistive force due to the dissipative elements in viscoelastic materials is expected to impact not only the size and maturity of the focal adhesions, but also many other downstream cellular processes [86].

It has been proposed that human MSCs adhered to viscoelastic substrates will attempt to restore the balance of the lost tension through alternative mechanisms, such as increased spread area (isotonic cytoskeletal tension) and increased cell-cell contact (passive tension), behaviors both associated with human MSC differentiation [12]. Cameron has confirmed this statement by showing that increasing the loss moduli of polyacrylamide (PAA) gels from 1 Pa to 130 Pa (with constant storage modulus, such that Young's modulus equals 13.5 kPa) increases human MSC spread area and proliferation but decrease the size and maturity of focal adhesions [12]. The ability of human MSCs to differentiate towards a number of lineages is also enhanced on substrates with high loss modulus.

The study also demonstrates that inhibition of isometric tension in human MSCs on high loss modulus substrates produces no significant changes in cell behavior, whereas inhibition of isotonic tension does, indicating that isotonic tension helps human MSCs to sense creep [12].

#### 2.2.2. Pliable (Viscoelastic) Materials Impose a Limit for Cell Traction

In their recent paper, Cameron et al. [86] have introduced the hypothesis that the traction forces applied by human MSCs on both high-creep and low-creep hydrogels with the equivalent storage (elastic) moduli will initially be equivalent. However, due to the time-dependent dissipation of energy in high-creep hydrogels, there will be a limit to the force that cells are able to exert at their focal adhesions on these substrates.

The analogous cell behavior has been observed by Pek et al. [87] on 3D thixotropic polyethylene glycol (PEG) silica gels that become liquid when applied shear stress exceeds certain threshold. Though, as in viscoelastic gels, there is a limit to the force that cells are able to exert at their focal adhesions on these substrates, cells have been shown to exert maximum force that is possible on given substrate. The highest expressions of neural, myogenic, and osteogenic transcription factors have been obtained for gels with liquefaction stress of 7, 25, and 75 Pa, respectively. These stresses can be interpreted as maximal traction forces applied by cells.

The polymer material with viscoelastic properties that is most frequently used in* in vitro* studies of stem cell differentiation depending on substrate stiffness is polydimethylsiloxane (PDMS) substrate [49, 88, 89]. Researchers have noticed that neural induction by soft (5 kPa) flat polydimethylsiloxane (PDMS) surfaces is not as drastic as that by pure elastic substrates [88]. Those results correlate with the main conclusions made by Cameron et al. [12]: loss component of viscoelastic modulus allows cells to exert less force and develop fewer adhesions. It is thus less permissive for neuronal differentiation, which probably requires stronger adhesions.

### 2.3. Pore Size, Porosity, and Permeability

Porosity is a parameter that refers to fraction of the gel volume filled with liquid phase, which is the volume of voids around matrix scaffold molecules per unit volume of the gel. Pore size is a very different parameter, which, unlike porosity, directly refers to geometry of pores. Cell adhesion and motility depend on size of the pores, rather than porosity. Mean pore size has correlation with porosity for many synthetic polymers of simple composition; however, for natural polymers like collagenous gels there is no direct correlation, since the diameter of collagen fibrils can vary from few nanometers to a few hundred nanometers [23].

Permeability of ECM defines accessibility of small molecules (nutrients, hormones, and oxygen), large molecules (e.g., that is function of basement membranes), cell processes (e.g., axons), or cells (vascularization). Permeability of ECM to cell migration is important for regenerative processes. Poor permeability for cells, such as in scarred tissues, results in poor regeneration. Permeability for fluid flows and molecular diffusion is important for cell survival, since low permeability may result in lack of nutrients and ischemia.

Matrix scaffolds act mostly as a mechanical hindrance for fluid flow, diffusion, and cell migration [90]. Electric forces or other minor factors can play a role in some cases. In general, stiffer matrices made of the same material have lower pore size and permeability. Polymer substrates of the same stiffness but variable pore sizes can be produced (see Table 3). It is important to note that solute permeability of the matrix is enhanced under dynamic deformations due to increased fluid flow [91]. Permeability for the cells also is affected by substrate stiffness and viscoelasticity, because cells can deform actively more pliable matrices to move through. These aspects are still to be investigated.

#### 2.3.1. Stem Cells and Pore Size

Decoupling the effects of pore size and elasticity in order to examine the contribution of individual cues is highly challenging [13]. Substrate porosity seems to be an important factor as it can vary the length between two adjacent anchoring points, to which cell can adhere [49]. For example, a microporous foam material, wherein the pore size greatly exceeds the cell, effectively presents a slightly curved substrate to the cell as it adheres to a scaffold. As the cell attaches only its basal surface to the material [92], mechanotransduction mechanisms may be similar to those on planar substrates. Microscopic pores of about cell-size lead to a low tension, more grounded cell contacts with the material in all dimensions. There may exist a gradual transition from first variant to the last for intermediate pore sizes [13].

Recently, Wen et al. [49] have shown that stem cell differentiation does not depend on porosity. Adipose stromal cells and MSCs have been plated onto 13 kPa and 30 kPa polyacrylamide (PAA) hydrogels. Porosity of the gels has been varied by changing the weight ratios of acrylamide monomer and bisacrylamide cross-linker while maintaining constant stiffness [49]. Resulting pore sizes have been ranging between 23 and 45 nm and between 88 and 166 nm, considerably less than the cell-size. Cells undergo osteogenic differentiation only on stiffer gels independent of porosity. This study confutes the previous investigation that tends to prove importance of porosity for stem cell differentiation [89].

In experiments by Peyton et al. [51], on MSCs motility in 3D scaffolds based on polyethylene glycol (PEG), pore diameter has been varied from 7 to 17 *μ*m (i.e., from significantly smaller than the spherical cell diameter to approximately cell diameter). Cell speed is the highest within larger pores, but net displacement of the cells within matrix is maximal for intermediate pore-sizes, probably because of difficulty in finding straight way in the large-pore scaffold.

### 2.4. Static and Dynamic Deformations of the Matrix

#### 2.4.1. Mechanisms by Which Deformation and Inner Tension in ECM Affects Remodeling of Tissues

*In vivo*, many tissues are subjected to static and dynamic impacts, such as pulsations and flow in blood vessels (especially arteries) and compression and stretching during muscle and joint movements. During both compression and stretching matrix molecules, mostly fibrils, become stressed: stretched, compressed, or bended, which we refer to here as tension. Because of the complex, scaffold-like structure of ECM, some molecules can be stretched due to compression of the whole tissue or, vice versa, be compressed during stretching of the whole tissue.

Though static or dynamic deformations are not exactly properties of the material itself, they have also to be considered as important parameters, since they can alter the elastic characteristics (Young's modulus) of ECM because of its nonlinear elasticity and can alter the viscoelastic characteristics of ECM because of time-dependent nature of viscoelasticity.

Certain tissues* in vivo* are normally subject to significant tensions or compressions, for instance, skin or cartilage [36]. Skin tension can be demonstrated by the observation that the skin relaxes to form a wound of a greater diameter than the incision. Circular wounds tend to elongate in the direction of the greatest stress, indicating anisotropic nature of tissue. When skin is stretched, for example, during growth of underlying tissue, the skin adapts to reduce this increase in mechanical tension by increasing its own mass, volume, and area by a process of growth [93]. Alike, deformations and inner tension in ECM affect remodeling of other tissues.

Matrix tension and deformation can affect cells in two different ways, first, through forces that surrounding molecules apply to the cell surface through pulling on integrins or pushing cell membrane, and thus changing cell shape [14, 73]. Also dynamic deformation of the porous matrix leads to the fluid flow through the pores and along the cells that impose shear stress to the cells [91]. And shear stress and fluid flow are shown to influence stem cell behavior [11]. For example, ESC subjected to fluid flow differentiate into vascular endothelial cells [94]. As a conclusion, forces exerted on the cells due to ECM deformations are important cues and can be tangential (e.g., shear stress in arteries) or normal (e.g., compression in cartilage and/or bones).

Opposed to this direct mechanism is the change in ECM material properties due to deformation. Elastic characteristics depend on strain magnitude. Usually biological tissue stiffens under strain (arterial wall as example [25, 95]). This change can be caused by fiber reorientation, fiber straightening, and stiffening, stretching of linking elements to their limit, fluid outflow, and so forth. The opposite also can take place: decrease in stiffness is seen before breaking at high strains; some gels show thixotropic behavior (it turns to liquid when threshold stress is applied, as, e.g., gelatinous gel) [23]. Possible cause of ECM softening can be fiber weakening after stretching, breaking of connections between scaffold molecules [23]. Viscoelastic characteristics depend on strain rate and consequently on frequency of dynamic deformations. The same material may behave either like a viscous fluid or like elastic material (with mild tendency to viscoelasticity) dependent on frequency of the cyclic load and relaxation time of the material.

Furthermore, tensions of ECM molecules can play essential roles in tissues remodeling. By stretching fibrils in the matrix scaffold deformation alters its elastic properties and ability to reorganize. For instance, matrix metalloproteinase-1 (MMP-1), the interstitial collagenase that plays role in certain regenerative processes, degrades collagen fibrils at 100 times higher rate if the fibrils are under physical stress [96].

Additionally, cells themselves apply force locally to a matrix through their adhesions. While deforming the surrounding matrix, cells experience a resistive force, which depends on the intrinsic mechanical properties of the matrix. Cell traction forces can induce physical unfolding of some ECM molecules, such as fibronectin and collagen [73], or stiffen the surrounding material by stretching molecular linkers [1]. This substrate-induced mechanical feedback also drives cellular behaviors. Consistently, cells experiencing higher tension are preferentially committed to the osteogenic lineage, whereas those in low-tension regions became adipocytes [97].

#### 2.4.2. Mechanical Induction of Stem Cell Differentiation

Mechanical deformations ultimately have to influence stem cell behavior, especially in case of tissue damage, to induce regeneration. It is established, from* in vivo* observations, that regeneration of tissues often is more effective when the damaged tissue is subjected to mechanical stimulation similar to what it is accustomed to before damage [14]. There exist examples of effective arterial wall [25], cartilage [26], bone [98], and tendon [99] regeneration under proper mechanical loading, which is partly due to proper stem cell differentiation.

In an attempt to analyze MSC differentiation in the deforming damaged tissue, computer analysis models have been developed, for example, a model by Prendergast et al. [100]. Following this model, granulation tissue that emerge between bone and implant will transform into fibrous connective tissue, fibrocartilage, or bone depending on dynamic clues: relative fluid velocity through matrix and shear strain. If the extent of shear strain and fluid flow rate would be too high, then MSC within granulation tissue would differentiate into fibroblasts. In case of moderate motion osteogenic and chondrogenic differentiation is possible. If no shear or flow will be present, then resorption takes place [14, 98]. This model is partly confirmed by experimental observations [101].

Several studies show the effect of the tissue mechanical deformation on stem cell behavior* in vitro* by applying stress to 2D or 3D substrates seeded by stem cells [14]. Static compression of substrate (of 20–30% strain) has been shown to stimulate chondrogenic differentiation of embryonic limb bud mesenchymal cells [102]. Tensile strain of substrate has been shown to promote tendon-specific differentiation of MSCs [99] and, according to other studies, enhance the expression of ligamentous, fibrogenic, osteogenic, and chondrogenic markers in MSCs [103].

Dynamic deformations have been also demonstrated to enhance chondrogenesis in studies involving different scaffolds and suggesting that cyclic compression alone is sufficient to induce chondrogenesis [14, 103, 104]. A study by Li et al. [105] has investigated a range of regimes of dynamic compression and shear stress that may drive differentiation of MSCs toward chondrogenic, osteogenic, or fibrous lineages. Authors have demonstrated that higher dynamic frequencies (1 Hz) and higher compression amplitudes (20%) induce the greatest chondrogenic gene expression, while lower amplitude (5%)/lower frequency (0.1 Hz) conditions induce a greater ratio of osteogenic markers to chondrogenic markers [105].

Application of cyclic tensile strain on MSCs typically leads to osteogenic differentiation if strain magnitudes range is from 0.4% to 5% [106]. Higher levels of tensile strain favor differentiation toward a tendon/ligament-type phenotype or myogenic differentiation [107].

In a study directly comparing cyclic compressive and tensile stimulus, stretching has been found to regulate many osteogenic and fibroblastic genes, while compression enhanced many chondrogenic-related genes [103]. Authors emphasize cell–matrix interactions in determining the response of MSCs to extrinsic mechanical cues such as dynamic compression and tensile deformation [103].

A study by Kurpinski et al. has demonstrated that imposing substrate strains of just 5% amplitude at 1 Hz frequency can induce MSCs differentiation toward smooth muscle [108]. Interestingly, the orientation of mechanical strain on MSCs and endothelial cells is proved to be important: proliferation is observed to be stimulated with mechanical strain of substrate along the axis of cell elongation but is not affected when the strain is perpendicular to it. And, on the other way around, cyclic uniaxial strain on elastic substrates causes the cells to align perpendicularly to the strain axis [17, 108]. Interesting effect has been achieved in this study by means of topographical patterning: the substrate with parallel microgrooves induces MSCs alignment parallel to the microgrooves. As a result, application of cyclic uniaxial strain then does not force them to change the orientation and MSCs remain aligned parallel to the strain axis.

## 3. Spatial Presentation of Epitopes

Physical characteristics of the matrix, such as stiffness, viscoelasticity, and plasticity, can be perceived by the cells due to establishment of adhesion contacts between the cells' receptors and ECM adhesive molecules. Biochemical aspects of ECM receptors interactions, such as affinity and specificity, are obviously significant (this matter will be discussed in Section 4). During the last decades, however, it has become evident that the same adhesion molecules, being spatially presented to the cell in a different manner, induce very distinct patterns of cell behavior.

**Figure 2:**
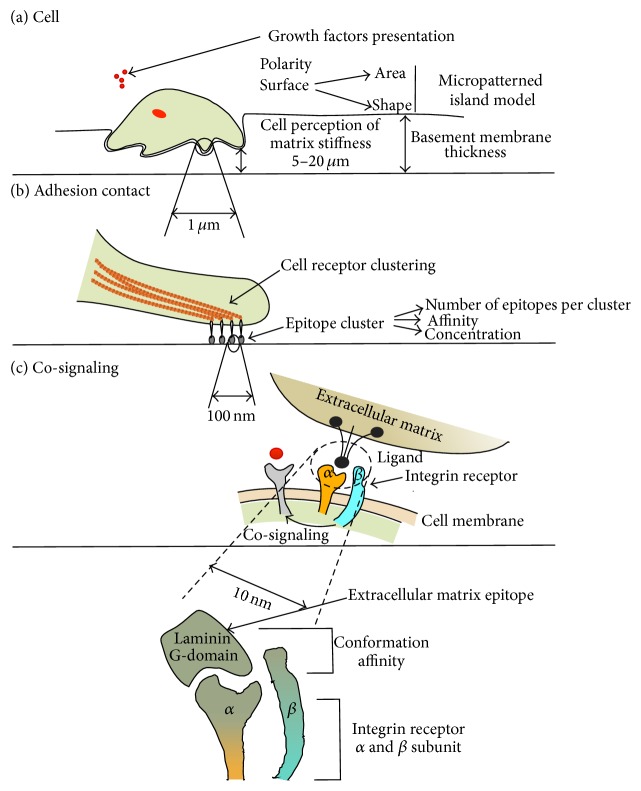
*Interactions of the cell structures with adhesive matrix epitopes: from cell-size scale to nanoscale*. (a) Certain events of matrix perception by the cell occur on scale comparable with size of the cell. Cells distinguish between 2D and 3D presentations of the epitopes. In case when 3D matrix is presented only from the basal side of the cell, thickness of the matrix layer can be critical for cell perception of the matrix. Cell perception of the underlying matrix thickness ranges approximately from 5 to 20 micrometers, which is comparable with average cell diameter. In cases when matrix defines the geometrical properties of cell adhesion surface, such as in case of adhesion islands model, adhesion area and shape can be influential factors for stem cell behavioral choices. (b) Cell propagates and spreads on the matrix due to spreading of filopodia, which form stable adhesion contacts with the matrix. In order to achieve stability of such adhesion contact, it takes not a single epitope but cluster of epitopes which, in turn, enable clustering of cell receptors and signaling to the cell. The following parameters of epitope clusters may be important to influence cell behavioral choices: number of epitopes per cluster, space between clusters, and so forth. Topography on micro- and nanoscale can also be influential on cell behavior. (c) Such events like co-signaling (synergy between the different cell receptors, i.e., receptor for matrix epitopes and receptor for growth factors) occur on even smaller scale. Importantly, the natural matrix adhesion molecules are very large ones (molecular weight up to 1 million Daltons, length up to 300 nanometers), compared to typical adhesion peptides (composed of several amino acids, with molecular weight below 1 thousand Daltons). Apparently, one has to consider geometry of functional clusters of receptors and matrix molecules on molecular level. (d) Affinity and specificity of matrix adhesion epitopes interactions with cell receptors occur on even smaller scale.

There are numerous characteristics that govern epitope presentation to the cell, including dimensionality (2D or 3D) of the scaffold; thickness of the substrate layer underlying the cell; cell polarity; surface area and geometry of adhesion surface; microscale topography of the surface; epitope concentration; epitope clustering characteristics (number of epitopes per cluster, spacing between epitopes within cluster, spacing between separate clusters, cluster patterns, and order or disorder in epitope arrangement); and size, shape, and level of disorder of nanotopographical features. It is important to consider the epitope presentation to the cell on different levels of magnification: matrix dimensionality, cell polarity, surface area, and shape are on the cell-size level, while distance between individual epitopes and nanotopography features are on the nanolevel (see Figure 2 for illustration).

### 3.1. Matrix Dimensionality and Dimensionality of Epitope Presentation to the Cells

#### 3.1.1. 2D Adhesive Culture Systems versus 3D Gels

Two-dimensional (2D) culture systems are the traditional, simple, convenient, and most frequently used systems for growing adhesive cells. In those systems adherent cells are spread in a monolayer upon a solid, impermeable surface, usually plastic or glass, and are exposed to culture medium from apical side. Discovery of the matrix adhesion molecules, such as fibronectin, laminin, vitronectin, and, in certain cases, specific collagen types, improved the cell culture systems greatly, by providing more stable adhesion and better cell spreading and providing vital adhesive contacts to the cells. Two-dimensional systems have many advantages, such as easy culturing techniques, such as culture medium replacement and cell passaging, easy cell assessment and quantification, high-quality imaging (including evaluation of minor cell organelles, such as mitochondria, vesicles, or cytoskeleton structures), possibility to run high-throughput imaging assays with high rate, and compatibility with robotic cell culture systems. Many studies aimed to investigate cell-cell and cell-matrix interactions are performed with cells seeded on rigid surfaces directly coated with various adhesive proteins [16, 109].

Three-dimensional (3D) cell culture systems, such as collagen gels, fibrin gels, or synthetic gels, are used to imitate soft and permeable tissues properties. Cultured cells are either immersed in the 3D gel completely or are cultured on top of the 3D gels. For instance, collagen gels or synthetic matrices are used as planar support for a cell monolayer in order to imitate ECM-like substrates about 100 *μ*m thick [10]. In the latter case the epitope presentation to the cell is 2D, despite the fact that the layer itself is composed of a 3D gel.

The 3D gels have higher biological relevance, compared to 2D-adherent cultures, in respect to the matrix physical properties and permeability; however, mere three-dimensionality is not a universal solution for all the mammalian cell cultures [16]. Cell-matrix adhesions in 3D environment (both* in vitro *and* in vivo*) involve the same molecules as in 2D cell contacts, integrins, vinculin, and paxillin, but they differ in spatial organization [110].

Porous and fibril-composed scaffolds are commonly perceived as 3D-carriers; however, cell perception of the scaffold depends on the pore size or fibril size. Notably, if the pores in the scaffold exceed the cell size, then cell behavior in such scaffold will be analogous to planar substrate, since cells are residing on the wall of the pore or the fibril and perceive it as a 2D surface [13].

#### 3.1.2. 2D-Like 3D Systems: Basement Membranes as* In Vivo* Example—Role of Gel-Like Substrate Thickness

Certain stem cell types naturally require 3D environment, wherein the adhesion contacts form from all sides of the cell. Examples are niches in bone marrow, brain, and muscle [111]. On the contrary, some other stem cell types require contact with 2D-like basement membranes (where the epitopes are presented from basal side only). Thus, for certain types of mammalian cells, including certain stem cell types, 3D presentation of matrix epitopes does not imitate features of their native niche. For example, endothelial and epithelial cells, as well as specific stem cell types like epithelial stem cells [79] or sperm stem cells [112],* in vivo* are adherent to basement membranes.

However,* in vivo* the cells do not perceive surface as impermeable underlay, such as* in vitro* culture plastic or glass surfaces. Basement membranes share certain features of 3D matrices (molecular organization of collagenous and laminin meshwork) and also features of 2D (such as epitopes presentation to the adherent cells from basal side only). Because basement membranes has certain thickness, which ranges from 50 to 100 nm for thin basement membranes to about 300 nm for kidney glomerular basement membrane [113], the cells can draw projections into it and perceive it as 3D matrix. As mentioned in the Section 2, cells can feel matrix layer underneath the substrate surface, to which they are attached (e.g., basement membrane) on distances from 5 *μ*m to 10–20 *μ*m. These values are considerably higher than basement membranes thickness range. Thus, the matrix under basement membrane* in vivo* is influencing cell behavior. Even deformation by cell traction can penetrate to this depth. As for the experimental setups, it should be concluded that the distances of micrometer scale from the cells to the constraints have to be considered.

#### 3.1.3. “Sandwich” 3D Systems and Stratified Scaffolds

Cells recognize matrix epitope presentation as 2D or 3D mainly on the basis of whether integrin-mediated adhesions to the ECM form on one face of the cell (basal) or all around the cell surface [16]. To capture the features that arise from the spatial distribution of adhesions in 3D environment the “sandwich” system has been developed. In the study of Beningo et al. in 2004 [114] fibroblasts have been placed between two ECM-coated polyacrylamide (PAA) gels, adhere to both surfaces, and develop stellate morphology with long actin-rich extensions similar to their shape* in vivo*.

Rehfeldt et al. have argued in their study [52] that overlays of matrix on 2D cultures are useful since stratified 3D microenvironments are evident in tissues such as the muscle stem cell niche. In these niches satellite cells reside between the muscle fiber and the basal lamina and experience distinct matrix compositions on their basal and apical surfaces. By modulating the stiffness of the matrices, authors have shown that the MSCs respond to that of two hyaluronic acid (HA) matrices, which is more rigid. The cells acquire elongated shape while driving cytoplasmic stress fiber assembly. In contrast, embedding cells in homogeneous hyaluronic acid (HA) matrices constrains cells to spherically symmetric shapes in which stiffness drives the assembly of a predominantly cortical cytoskeleton.

#### 3.1.4. Effect of Matrix Dimensionality on* In Vitro *Stem Cell Cultures

Both 2D and 3D ECM systems are successfully employed in stem cells* in vitro* culture systems. Hereby, we present several examples of how the difference between 2D and 3D environment may affect stem cells behavior.

Murine ESC-derived embryoid bodies maintained in 3D culture in polyethylene glycol- (PEG-) based hydrogels have shown significant upregulation of cartilage-relevant markers, as compared to a monolayer culture system [18]. Rat bone marrow-derived MSCs show a 3.5-fold increase in percentage of insulin-producing cells in 3D experimental group compared to 2D culture cells. They form spherical-type agglomerates with confluence in 2D environment but show round-shaped morphology in 3D environment [115]. Patterns of murine ESC differentiation into neural and glial lineages in 3D scaffolds are significantly different from those on 2D coated substrates. A twofold increase in neural differentiation and fourfold increase in formation of astrocytes have been observed in gelatin 3D gels compared to the 2D. A twofold increase in astrocyte differentiation and 6-fold increase in oligodendrocyte formation have been observed in collagen 3D gels compared to the 2D [116]. Differentiated human neural stem cells cultured in an inert 3D scaffold form spontaneously active, functional neuronal networks in contrast to 2D cultures [117]. Differentiation of human ESC to dopamine neurons in 3D alginate microcapsules is more efficient than in 2D cultures: neuronal markers expression increases by >100-fold [118]. MSCs encapsulated within the gel modify from rounded morphology in a densely cross-linked gel to a spread shaped form by reducing the cross-linking density of the gel in a photodegradable polyethylene glycol- (PEG-) based scaffolds [119].

In summary, 3D scaffold appears to be more appropriate for neuronal differentiation and formation of neural network. Culturing in 3D environment induces a more rounded, spheroidal cell morphology in comparison to standard 2D culture systems that favor cells spreading and flattening [18].

Difference of 3D and 2D substrates regarding effect on stem cell fate on macrolevel is a consequence of the several differences between 3D systems and 2D systems. First of all, most of living tissues are characterized by low stiffness (exceptions are bone, tendon, and cartilage), but traditional 2D culture on glass or plastic substrates places cells in a static mechanical environment that is up to a billion times stiffer than physiological milieu. Second, the difference observed between cells that were cultured in 2D versus 3D environments is dissimilarity in morphology (cell shape, polarity). Cells grown in a monolayer are flat and can adhere and spread freely in the horizontal plane but have no support for spreading in the vertical dimension. Third, most of soluble ligands (growth factors) cannot diffuse freely in 3D ECM, because they could be captured by ECM components (e.g., fibronectin, glycosaminoglycans, hyaluronic acid, and others) and therefore can be presented by ECM to cells in a complex with these components.

### 3.2. Cell Polarity and Micropatterned Adhesion Islands

Cell polarity refers to spatial difference in the cell arrangement, such as geometrical shape, structure, and niche contacts. Majority of mammalian cell types exhibit certain polarity, dependent on native niche and function. Examples of polarity are apical-basal polarity in cells residing on basement membrane (epithelial cells, satellite cells), polarity of neuron required to provide directional signal transduction, and polarity of migrating cells with development of lamellipodia or filopodia at the leading edge.

Adherent cells cultured as monolayer undergo a forced apical-basal polarization. They appear to have distinct “apical membrane” facing lumen filled with liquid solution and the “basolateral membrane” oriented downwards to the surface and laterally to the other cells. Adhesion is provided by adhesion contacts developed on basal side of the cell membrane, which is attached to basement membrane* in vivo* or to artificial substrate* in vitro*. Apical-basal polarity is relevant for certain cell types, such as basement membrane-adhered cells, but is unnatural for most mesenchymal cells, which spread in all directions in 3-dimensionality and only polarize from front to rear during migration [120]. Apical-basal polarity can also impact signal propagation inside the cell and modulate the sensitivity of cells to apoptosis [16]. Receptors of polarized cell can be localized on basal side, which can be a cause of cell malfunction in 2D cultures. That has been demonstrated on mammary epithelial cells grown on 2D substrates. They fail to undergo functional differentiation in the presence of the lactogenic hormone (prolactin), because receptors for the hormone are localized on the basolateral cell membrane and therefore not accessible to its ligand [121]. When the polarity is reverted and the hormone is presented from the apical side, the prolactin-driven expression of *β*-casein is restored.

#### 3.2.1. Basal Cell Surface Area and Shape: Effect on Cells Behavior* In Vitro*—Micropatterned Islands Approach in Generation of Artificial Scaffolds That Can Control Cell Surface Area

Cell behavior can be affected by forced modulation of adherent (basal) cell surface area and shape. Such results are achieved by method of micropatterning, which enables formation of the highly adhesive regions of particular size and shape on artificially fabricated planar surfaces that are termed micropatterned islands. The size of the* in vivo* microenvironment usually limits the cell volume and spreading area. Spatial distributions of cell adhesion and that of unattached cell surfaces are dictated by location and orientation of ECM fibers, pores, and other cells [17]. Micropatterned single-cell substrate islands allow the reconstitution of tissue-like conditions* in vitro* by imposing a defined cell attachment pattern by engineered constraints and microscopic features [17]. For methods of micropatterning, address to the excellent review by Théry [17].

By using the method of micropatterned adhesive islands, it has been demonstrated that altering the degree of cell spreading can impact cell proliferation, apoptosis, and differentiation [122]. On small ECM micropatterned islands cells adopted in general a poorly spread rounded morphology, whereas cells adhered to large ECM islands adopted flattened morphologies typical of 2D cultures [18].

Differentiation of stem cells is guided by micropatterning through the same mechanisms as substrate stiffness does, for example, by dictating the level of cell contraction, which increases with cell spreading. Larger size or elongated shape of an island forces cells to spread more. For example, individual human MSCs cultured in differentiating medium and plated on 1000 *μ*m^2^ square islands, made of polydimethylsiloxane (PDMS) substrate coated by fibronectin, differentiate into adipocytes, whereas those plated on 10,000 *μ*m^2^ micropatterns differentiate into osteoblasts [82]. Under the treatment by transforming growth factor *β* (TGF-*β*), human MSCs differentiate into chondrocytes on small (1000 *μ*m^2^) fibronectin-coated islands but into myocytes when seeded on large (10,000 *μ*m^2^) islands [123].

The stem cell fate depends not only on the extent of cell spreading but also on the convexity of cell edges. Consequently, human MSCs grown on convex geometries, such as a pentagon-shaped micropattern, differentiate preferentially into adipocytes, whereas those grown on concave geometries, such as a star-shaped micropattern, tend to differentiate into osteoblasts [124]. In these experiments, shapes with areas of 1000, 2500, and 5000 *μ*m^2^ have been used.

Interestingly, it has been recently proved for MSC on polyethylene glycol (PEG) hydrogels of 1–20 kPa that on small adhesive areas size of an island is the most significant physical signal for lineage commitment. Cultured on 1000 *μ*m^2^ circles, squares, and rectangles, MSCs undergo primarily adipogenesis regardless of matrix elasticity. At larger adhesive areas shape and matrix elasticity are becoming significant. These parameters are shown to guide differentiation of cells cultured on 2500 and 5000 *μ*m^2^ shapes [48]. Accordingly, epidermal stem cells on small (20 *μ*m diameter) circular islands are shown to remain rounded and terminally differentiate to keratinocytes at higher frequency than cells that are able to spread on large (50 *μ*m diameter) islands [122].

The micropatterned islands techniques have been used to define how the extracellular environment affects cell polarity, defined by the cortical and internal cell asymmetry [125]. In the study has been analyzed the organization of individual cells plated on defined 2D micropatterned substrates imposing cells to have identical square shapes, but on various adhesive areas (X, C, K, and arrow-shaped islands having similar square convex envelops). The internal polarity of human retinal pigment epithelial cells plated on these fibronectin micropatterns has been shown to depend on the geometry of the adhesive surface, as judged by the position of the centrosome with respect to the nucleus.

The different kind of micropatterns that provide topographic pattern to the cells, such as grooves of about cell-size width, is also tested in several studies [54, 108, 126]. It is shown that MSCs align with fabricated microgrooves (10 *μ*m in width, 3 *μ*m in height) via contact guidance [108]. Also surface microtopography is a critical factor for manipulating stemness of ESC as shown using groove, pillar, and hexagonal polyacrylamide (PAA) substrate (with size of obstacles about 5 *μ*m) [126]. It has been concluded that although topography is less influential when cells are cultured upon soft substrates, it plays a significant role in retaining cell stemness on stiff hexagonal or pillar-shaped substrates. Similar results are observed for the rat MSCs [54]. As mechanisms of topography sensing by cells on microscale are probably the same as on nanoscale [108], further information on the subject will be provided in nanotopography section.

Despite the apparent role of adhesive shape and area on cell function in 2D culture, it remains unclear how these insights map to 3D settings [16].

### 3.3. Epitopes Arrangement in Clusters

#### 3.3.1. Formation of Stable Cell Adhesions Involves Grouping of Integrins, Which Depends on Epitope Organization

In the previous section, the adhesive epitope-bearing scaffold or ECM has been regarded as continuous material coated homogeneously by excessive amount of adhesive proteins. The question arises as to what is the density of adhesive sites threshold that distinguishes adhesive surface from non-adhesive one? Now we shall consider ECM perception by the cells on the scale of ECM receptors, which are the means of cell attachment to adhesive matrix proteins. We describe studies of cell attachment to substrate that consider distinct adhesion sites and how inhomogeneity can influence cell behavior.

Cells have the ability to sense micro- and even nanoscale geometric cues from their environment. This is possible through sensing of epitopes (or insoluble ligands), parts of proteins or peptides that are attached to matrix scaffold and that have affinity to specific cell receptors, for example, integrins. To form an adequate adhesion that is able to transmit force, several adjacent integrins are required to be grouped together. Gathering of integrins, made by contractions inside the cell, depends on distance between involved integrins [2], though distribution of epitopes plays a crucial role in cell adhesion.

Synthetic polymer materials used in* in vitro* experiments allow variations of such parameters of epitope presentation to the cell as epitope density, spacing between epitopes, epitope clustering, and surface nanotopography. The most abundant types of epitopes (or ligands) that have been studied in many works are RGD (from fibronectin, vitronectin), YIGSR and IKVAV (from laminin), and VPGIG (from elastin) [127]. RGD is a tripeptide composed of amino acids Arg-Gly-Asp. In various types of ECM, it is the prevailing adhesive ligand; therefore, binding of cells is dependent on RGD density and organization [127].

#### 3.3.2. Epitope Density and Spacing between Epitope Molecules

Epitopes concentration, or epitope density, is a parameter that may significantly affect cell behavior, including stem cell differentiation. In their studies Cavalcanti-Adam et al. have investigated effect of integrin-adhesive RGD ligands concentration per unit area on cell behavior [128]. When RGD epitopes are localized at high densities, so that neighboring RGD molecules are less than 70 nm apart, the fibroblast cells form focal adhesions and polymerization of contractile actin cytoskeletal stress fibers is observed. When the RGD ligands are presented at a lower density (more than 70 nm separation), low cell adhesion is observed leading to smaller focal adhesions and more rounded cells. Low adhesion can potentially lead to cell quiescence or to anoikis-programmed cell death due to “homelessness” [2]. A study on MSCs also confirms the hypothesis that if individual RGD ligands are presented at a density just over 70 nm apart, MSCs fail to group individual integrins into mature adhesions [129]. It is worth mentioning that introducing disorder to RGDs placed 70 nm apart allowed much greater integrin clustering in MSCs, as have been shown in the same study [129].

Arnold et al. have obtained similar results using 8 nm gold particles coated with RGD peptides that permit binding of only a single integrin. The authors have determined that the ligand spacing in the range between 58 and 73 nm is required for integrin-clustering-induced signaling to occur in osteoblasts, fibroblasts, and melanocytes. A separation of epitopes by 73 nm between the adhesive dots dramatically reduces the formation of focal adhesions and actin stress fibers, which results in limited cell attachment and spreading [130].

Results of the study by Silva et al. on neural progenitor cells cultured on nanofibers with IKVAV epitope suggest that high density of available epitopes promotes cells differentiation either in 2D or 3D cultures (7.1*∗*10^14^ IKVAV epitopes/cm^2^). In this study epitopes are presented by nanofibers at a roughly 1000 times higher density than by laminin molecules closely packed in a 2D lattice. Authors have concluded that density rather than dimensionality of epitope presentation is the key factor in the rapid and selective differentiation of cells into neurons [131].

#### 3.3.3. Cluster-Like Arrangement of Epitopes: Number of Epitopes Per Cluster and Space between Clusters

*In vivo* matrix ligands are sometimes arranged not as evenly spread single molecules, but rather in form of clusters. Such arrangement can be characterized by several parameters, such as number of epitopes per cluster, space between the clusters, size of clusters, and patterns that clusters form. It is accepted that clusters of epitopes result in grouping of the ligand-bound integrins more effectively than the same surface density of evenly distributed epitopes [2, 18].

There is evidence that in 3D matrix MSCs tend to foster RGD clustering by imposing traction and reorganizing matrix [132]. RGD clustering is maximized in matrices of intermediate rigidity. The result is consistent with previous data. The cells cultured on highly compliant substrates cannot assemble the cytoskeleton-associated adhesion complexes required to exert significant traction forces, whereas on very rigid substrates the cells cannot generate force sufficient to deform the matrix [132].

Several studies have been performed to measure the thresholds for epitope clusters size and spacing that allow sufficient cell adhesion [2, 133]. An approach applied by Schvartzman et al. [134] elicits the importance of adhesion gathering of at least four epitopes per cluster. In this study the arrays of RGD clusters are made using electron-beam lithography. The arrays contain clusters of two to seven RGD ligands (individual RGD units <60 nm apart), forming adhesive islands with approximately 200 nm spacing between the clusters (to prevent integrin clustering in the space between the clusters). Spacing, density, and cluster size of individual integrin binding sites are systematically varied. Cell spreading observation on arrays of different geometric arrangements reveal an increase in spreading efficiency when at least four ligand sites are spaced within 60 nm or less, with no dependence on overall density [2, 134].

Another study has examined the effect of both spacing and density of YGRGD epitope on fibroblasts [135]. A non-adhesive polyethylene oxide (PEO) hydrogel and PEO tethers have been used for making clusters of one to nine YGRGD adhesion ligands (attached to a star-like 6 nm wide PEO molecule). Distances between clusters have been defined in a range of 6–300 nm. In case of clustered ligands there are more adhered cells, higher percentage of cells with stress fibers, and enhanced cell motility. Importantly, even at the maximal density of individual YGRGD (30,000 epitopes/*μ*m^2^, spaced by only 6 nm), cell response is significantly lower than for clusters of nine YGRGD peptides with a density of 2,300 epitopes/*μ*m^2^ (190 nm between clusters) [2]. This study shows that critical distances are required to enable the cells to spread: a distance of 60 nm is necessary for clusters of nine peptide epitopes and 9 nm is needed for clusters of five epitopes, with <6 nm required for individually presented YGRGD molecules [2]. Authors conclude that there exist a minimum threshold cluster size and a minimum number of clusters and that both are required to achieve significant values of adhesion and migration.

Cell behavior can be also influenced by the coexistence of multiple peptides. For example, RGD and the site Pro-His-Ser-Arg-Asn (PHSRN), spaced by 4 nm, result in an increase in markers of osteoblastic cell function [127].

Direct comparisons between different epitope presentation systems are challenging because of alterations in concentration, spacing, or rigidity of the substrate and affinity of integrins to the epitopes. As matrix stiffness is proved to affect significantly formation of focal adhesions, it is crucial to investigate the epitope cluster effect with respect to substrate stiffness.

### 3.4. Nanotopography

Interestingly, the cells are able to recognize nanoscale topographical features. For instance, cells are able to align along collagen fibers* in vivo* [108]. Nanotopographical methods are developed to investigate influence on the cell fate of such geometric cues such as surface roughness (grooves, pits, or pillars) or fiber diameter.

It has been observed that human MSCs grown on nanoscale grooves of 350 nm width show alignment of their cytoskeleton and nuclei of MSCs along the grooves [136]. Polydimethylsiloxane (PDMS) gratings with 600 nm features and spacing are found to induce the alignment and elongation of ESC [18]. Mechanism of such sensing at nanoscale level is believed to be due to the clustering of integrins and other cell adhesion molecules because roughness of the surface provides particular spacing of adhesive sites [18]. When nanoscale grooves are used to guide cells, the initial responders aligning along the grooves are filopodia, fine integrin-containing cell membrane projections with a tip diameter on the nanoscale. The minimal height threshold at which substrate nanogroove dimensions may influence filopodial guidance and subsequent whole-cell alignment is around 35 nm [2]. Elongation and spreading of stem cells guided by nanotopographical clues control differentiation: it has been demonstrated that patterns with feature diameters of 100 nm can be used to control MSC osteogenesis with efficacy comparable to chemical stimulation [137, 138].

Furthermore, it has been shown recently that a disordered surface topography is capable of influencing the human ESC to differentiate towards a stromal osteoblast phenotype [139]. Earlier Dalby et al. [138] have demonstrated that nanoscale pits arranged with high level of disorder stimulate MSCs to produce bone mineral* in vitro*, in the absence of osteogenic media. This finding is consistent with an evidence that type X collagen molecules form disordered hexagonal structures of nanometer scale at sites of endochondral ossification in cartilage (mineralization of cartilage to bone) and at sites of large fractures [140], which may foster MSC differentiation to osteoblasts in these sites [141].

There is an interesting observation that nanotopographical periodical structures are important in MSC differentiation. Helical self-assembling amphiphilic molecules with a 63 nm periodicity have been shown to promote osteogenic differentiation of MSCs. This periodicity is very close to that of type I collagen fibers, about 67 nm, which is main protein composing bone. Notably, if the pattern periodicity is different from that of collagen, 100 nm, the osteoinductive effect decreases [142].

Thus, nanotopography provides a useful tool to control adhesions of stem cells and consequently to control self-renewal or differentiation status.

## 4. Molecular Complexity of Natural Matrix Epitopes

In Sections 2 and 3, we discussed how cells perceive the physical properties of matrix via binding adhesive epitopes and the importance of spatial arrangements of those epitopes without relating to biochemical nature of such adhesive interactions. Most experimental models used to research effect of ECM physical and spatial properties on cells involved very few types of short peptides, such as RGD or IKVAV, that are known to provide strong adhesion contacts. However, natural matrix adhesion molecules are numerous, complex, and often indispensable for healthy organism maintenance, as demonstrated by knockout animal models. Adhesive ECM molecules have developed very early in course of evolution. Already in simplest organisms like hydra they already exist as large and complex molecules and are essential for healthy maintenance and regeneration. It clearly appears that there should be biological reasons for variety and structural complexity of natural matrix molecules.

As the cell perceives the adhesion contacts via specific receptors (e.g., integrin receptors), signals that come from different receptors possibly cause different response [143]. However, concept of signaling transmitted by adhesion molecules of the ECM is not as simple as mere “pulling specific receptors.” Studies of niche-specific ECM ligands that provide adhesion contacts with cell receptors revealed the following: (1) different matrix adhesion molecules determine different patterns of cell behavior; (2) simplified concept of “pulling specific integrin receptors” cannot explain the effects produced by ECM on cells; (3) adhesive epitopes of ECM ligands are not exclusive effectors, and non-adhesive domains may influence the ECM molecule effect on the cell.

We shall discuss several paradigms, such as (1) co-signaling of ECM with growth factors receptors, cell-cell contacts, or other ECM receptors, (2) role of ECM as depot for growth factors and presentation of accumulated growth factors to the cells, (3) ability of natural large ECM molecules to serve as dual signaling hub and thus being able to bind two different cell receptors, and (4) importance of non-adhesive elements of large adhesion molecules. We shall often refer to laminins, a family of cell-adhesive ECM proteins, since it is well studied and can provide excellent examples to paradigms we discuss. The paradigms as such, however, may relate not only to laminins but also to other cell-adhesive ECM proteins.

### 4.1. Extracellular Matrix* In Vivo*: Supramolecular Architecture and Molecular Diversity

Over 40 years ago several adhesion molecules (fibronectin, vitronectin, laminin-111, or placenta-derived mixture of laminins) were discovered and successfully implemented to improve cell cultures* in vitro* [3, 4]. During the past years, the concept of adhesive matrix molecules developed and became far more sophisticated. It has been understood that adhesion is not only a convenient tool that allows keeping cells in place, but it may be crucial for survival and maintenance. Cells lacking their natural ECM cues undergo controlled suicide termed “anoikis,” which is a part of natural mechanisms that allows controlling proper compartmentalization of specific cell types. Malignant cells often start producing their own ECM molecules, which is crucial for tumor development and metastasis [144]. It has been shown that ECM molecules are not “neutrally” adhesive (i.e., generally good for culturing), but they send specific messages to the cells. Moreover, two similar molecules that provide similar “good adhesion” to the same cell may have antagonistic influence on the cell behavior. There is evidence that improved adhesion, survival, and proliferation can be not necessarily beneficial, but sometimes an indicator of early malignant transformation.

A variety of niche-specific cell adhesion ECM molecules have been discovered. “Laminin” turned out to be not just a single basement membrane-associated protein but represents at least 15 different isoforms with unique functions. Several different laminin isoforms were shown to exert clearly distinct, sometimes even antagonistic effects on cells. Biological function of many laminin isoforms is still poorly studied, though severe knockout phenotypes suggest them to be essential for certain mammalian cell types.

#### 4.1.1. Supramolecular Architecture of Architectural Matrix

Artificial scaffolds traditionally used for mechanoelasticity and epitope localization studies usually consist of very few functional elements (fibrils, cross-linking elements, and adhesive epitopes). The natural scaffolds of mammalian tissues are far more complex and consist of variety of molecules. For instance, a dozen of different collagen types, including fibrillar, fibril-associated, basement membrane-associated, and cell-associated types, are required to build the collagen scaffold of functional arterial wall of mammalian organism [95]. Collagenous scaffold is supplemented by miscellaneous non-collagenous molecules, like elastin, laminin-411, laminin-511, and many others. Knockout models in mice demonstrate that many of the structural ECM molecules have unique biological roles and are indispensable. Complexity of supramolecular organization allows achieving a range of physical characteristics, for instance, highly nonlinear stress-strain curve, that allows the arterial wall to maintain function through lifetime sustaining 2 billion pulsations of blood pressure with minor deterioration [25, 95].

#### 4.1.2. Diversity of Matrix Molecules

Collagens, laminins, fibronectin, vitronectin, nidogens, agrin, perlecan, netrins, nephronectin, and usherin are well-known ECM molecules [113, 145]. However, biological function and biochemical properties for many other ECM proteins are rather poorly studied.

Project Matrisome, initiated by Richard Hynes team, aimed to identify and measure all the ECM proteins present in mammalian tissues [146]. Up to date the project lists 44 collagens chains, 195 glycoproteins, and 35 proteoglycans in human proteome. Revealing the biological functions and biochemical features of all those proteins may allow advances in reconstructing biologically relevant niches for cultured cells* in vitro.*

#### 4.1.3. Versatile Functions of Natural Matrix Molecules

There is a tendency to regard certain ECM proteins either as cell-binding (and thus active effectors of cell behavior) or as mere structural. For instance, laminin family is traditionally known as the matrix molecules that directly enable cell signaling, since every laminin molecule has the G-domain of an alpha chain that binds to several integrin receptors. On the contrary, collagens are traditionally regarded as mere “structural” proteins, due to unique long-term stability and exceptional mechanical durability of collagen fibrils, composed of collagens I, II, III, and V. However, roles of those molecules can be versatile. Laminins also initiate the basement membrane formation; they build the primary scaffold, while collagen IV meshwork adds to it later [147, 148]. Collagens can signal to the cells by interacting with integrin receptors, for instance, integrins *α*1*β*1, *α*2*β*1, *α*10*β*1, and *α*11*β*1 [143].

#### 4.1.4. Natural ECM Molecules in Stem Cell Cultures* In Vitro* and Stem Cell Niches* In Vivo*

Natural ECM molecules proved to be highly effective in developing stem cell culture systems. For ESC culture expansion (self-renewal) fibronectin, vitronectin, laminin-111, and laminin-111 containing basement membrane extract named Matrigel, laminin-511 and laminin-521 were successfully implemented [149–153]. Though all those molecules were generally supportive for the ESC culture, they acted through activation of different signaling pathways and thus exerted distinct influence on cultured ESC. Notably, not all of the ECM molecules were biologically relevant for the purpose.

Laminins are the very first ECM molecules in the mammalian embryo; expression of laminin chains starts already at 2–4 cell embryo stages. Both laminin-111 and laminin-511/521 are present in mammalian blastocyst and are in contact with blastocyst inner cell mass cells, the* in vivo* analogue of ESC. However, the biological roles of those biologically relevant laminins are distinct and even antagonistic: laminin-511/521 is expressed by the inner cell mass cells and supports their self-renewal and maintenance [154, 155], while laminin-111 is expressed by trophoblast and it induces inner cell mass polarization, a first step towards differentiation into endo-, meso-, and ectoderm (reviewed in [7]).

In further chapters, we shall present more examples of biologically relevant ECM molecules being efficient scaffolds for culturing different stem cell types* in vitro*.

#### 4.1.5. Experimental Approaches to Investigate Effect of Biochemical Complexity of ECM Molecules on Stem Cell Behavior

As we discussed previously there must be strong functional reasons for ECM molecules to develop during evolution not as small adhesive peptides but as extremely large and complex molecules. There must be additional roles for the natural ECM molecules apart from providing mere adhesion contacts, a function for which short peptides of only 3–5 amino acids would be sufficient. However, the experimental approaches that would allow investigating specific roles for isolated structural elements of large and complex ECM molecules require advanced techniques, possibility to eliminate, mutate, or replace certain functional domains without disrupting the natural organization of the rest of the molecule. Use of recombinant techniques that would provide the partially modified molecules has a possible problem that unnatural modification of the molecule would impede the correct folding of the whole molecule and thus affect the functionality of the other domains. However, there is a useful tool to investigate molecular versatility of large ECM molecules, while working with natural ECM molecules: working with a family of ECM molecules composed of several heterogeneous chains and comparing two similar molecules which share most of the chains and domains but having distinction in other chains or domains. Laminin family is a well-studied family of ECM molecules that allows such versatility; also, certain laminin isoforms are the biologically relevant adhesion contacts for many stem cell types* in vivo* and have proved to be efficient for culturing stem cells* in vitro.*

#### 4.1.6. Laminins Family: A Versatile Model System to Illustrate Paradigms Related to Molecular Complexity of ECM Molecules

Laminin family, due to well-characterized heterotrimeric organization and domain structure, can serve as a convenient and versatile model to illustrate several principles regarding the role of affinity domains, selectivity of cell receptor binding, role of non-affinity (structural) domains, possibility of one complex molecule to serve a dual signaling hub, co-signaling with growth factor receptors, and so forth. Knowledge about biological function of certain chains and even domains comes not only from* in vitro* studies but also from knockout phenotypes in several species, such as mice, zebrafish,* Drosophila* fruit fly,* C. elegans*, and* Hydra*. Additional data has been obtained from more sophisticated models, such as conditional knockout models restricted to certain tissues and developmental stages. Some of paradigms, proposed for laminin research field, may as well be applicable to other adhesion ECM molecules, such as collagens VII, XVII, and XIII, fibronectin, and vitronectin.

#### 4.1.7. Laminins Are a Large Family of at Least 16 Tissue-Specific Isoforms That Mediate Cell Maintenance and Behavior

**Figure 3:**
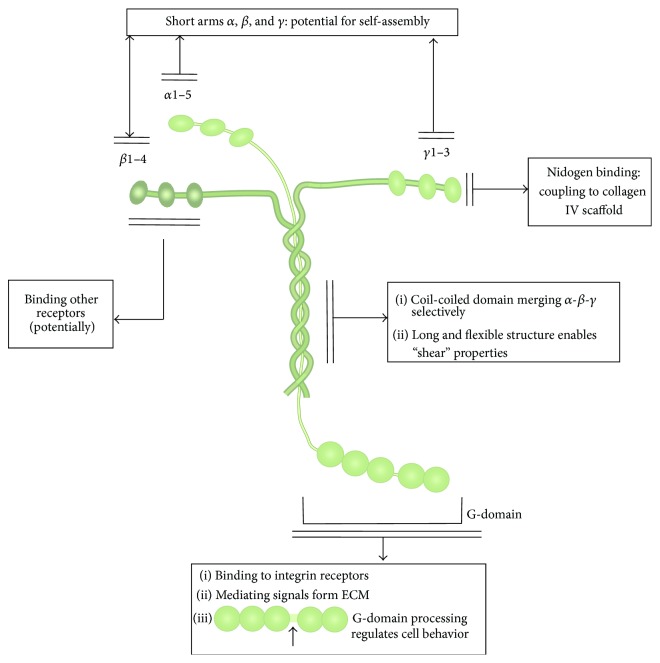
*Laminins: a model system to investigate roles of molecular complexity of natural matrix molecules*. Natural extracellular matrix molecules are very large and complex molecules, often composed of more than one polypeptide chain (collagens have 3 chains, laminins have 3 chains, and fibronectin has 2 chains) and multiple domains with distinct adhesive and geometrical properties. Mutations in either chains or domains often result in severe pathology. Apparently, such molecular complexity developed during evolution for a reason, and natural ECM molecules have more function, compared to small adhesive peptides. However, it is not easy to establish roles for each specific domain and structure of the natural large ECM molecules. Family of laminins, due to their molecular versatility, is a perfect model system to investigate reasons for functional complexity of matrix molecules. Laminins are the natural adhesion ligands for many stem cell types, like embryonic stem cells (ESC), hematopoietic stem cells, sperm stem cells, and probably many others. Due to unique composition of three heterogeneous chains, each of which can be varied, cell adhesion domains undergoing natural proteolytic maturation and, therefore, change in affinity and specificity, availability of conditional knockout models, and mutated proteins in laminin family are an excellent system to investigate reasons for molecular complexity of natural matrix molecules. Laminins are large, heterotrimeric molecules that comprise one *α*, one *β*, and one *γ* chain. Size of laminin trimer varies from 400 to 1000 kDa. Five *α* (*α*1–*α*5), four *β* (*β*1–*β*4), and three *γ* (*γ*1–*γ*3) chains are known in mammals. Laminin-521 (LM-521) consists of *α*5, *β*2, and *γ*1 chains. The molecule in the figure represents the cross-shaped laminin isoform; however, some laminin isoforms have truncated shapes: Y-like or rod-like shape. The *α*1, *α*2, *α*3B, and *α*5 trimers are cross-shaped, while the *α*3A and *α*4 trimers are Y-shaped or rod-shaped. Short arms of laminins (N-terminal parts of *α*, *β*, and *γ* chains) can bind other laminins short arms and other ECM proteins. Laminins in solution are capable of self-assembly via N-terminal short arms in the presence of calcium.

Laminins (LM) are large (500–1000 kDa) heterotrimeric cross-shaped molecules that convey ECM cues via cell receptors to cell signaling systems (Figure 3) and thus modulate behavior of associated cells, such as survival, adhesion, migration, proliferation, phenotype maintenance, or differentiation. It appears that most of the laminin isoforms have unique biological function that cannot be compensated by other, even highly similar laminin isoforms. Mutations in laminin-encoding genes often result in severe pathologies or lethality in most of the animal species, from mammals (like human or mouse) to the simplest organisms (like fruit fly,* C. elegans*, or even* Hydra*). In spite of molecular structure similarity and evolutionary homology certain laminin isoforms may exert antagonistic effects on cell behavioral patterns [7].

Certain laminins were demonstrated to act in concert with specific growth factors and cell-cell contact molecules, such as E-cadherin [153, 156]. Such synergy allows the long-term function of the niche-sensitive cells within the natural niches* in vivo*. Lack of either component in the niche system or replacement with similar, but not biologically relevant, molecule, may result in loss of function or stability in the cultured cells.

If a niche-sensitive cell is isolated from a natural niche environment and becomes devoid of biologically relevant laminin contact, it may undergo cell apoptosis, phenotype loss, or malignant transformation. Survival pathways for majority of mammalian cells depend on niche-specific ECM anchorage [8, 9]. Loss of such anchorage or irrelevant anchorage may activate the apoptotic pathways and results in apoptosis, which in this case is termed “anoikis.” Otherwise, it may activate malignant pathways of anchorage-independent antiapoptotic signaling. Thus, niche-specific laminins are essential for healthy development and maintenance of multiple mammalian cell types, such as ESC [151–153, 155], insulin-producing pancreatic *β*-cells [157], neural cells [158–160], and many other cell types (reviewed in [7]).

#### 4.1.8. Examples of Stem Cell Types Dependent on Biologically Relevant Laminins

*Embryonic Stem Cell Niche In Vivo and In Vitro*. LM-511/521 and LM-111 have similar structure; however, they have very distinct effects on cultured pluripotent stem cells. As we have discussed above, LM-111 causes polarization of blastocyst inner cell mass cells (the* in vivo* analogue of ESC), which is followed by differentiation of the cells into endo-, meso-, and ectoderm [161–163]. LM-511/521, on the contrary, stimulates self-renewal of inner cell mass cells* in vivo* and ESC* in vitro*. The* in vitro* use of LM-511/521 allows supporting stable self-renewal of mouse and human ESC for months, while combination of LM-521 with cell-cell adhesion molecule E-cadherin allows self-renewal even for ESC isolated from cell-cell contacts [151–153, 155].

*Sperm Stem Cells Niche In Vivo*. Many adult stem cells* in vivo* reside in specific niches that are located in proximity to the basement membranes. However, roles of specific laminin isoforms, present in those basement membranes, remain mostly unclear. Sperm stem cells* in vivo* reside in direct contact with the local basement membrane that contains LM-213. The cells maintain stemness while residing on the basement membrane; however, their detachment from the basement membrane correlates with gradual differentiation. A specific mutation causing laminin *α*2 chain deficiency caused concurrent reduction of laminin *γ*3 chain and abnormalities in testicular basement membranes [112]. LM *α*1 chain overexpression can compensate for lack of *α*2 in LAMA2 mutant mice and partially rescue the infertility caused by the *α*2 deficiency [112]. Notably, laminins *α*1 and *α*2 chains are evolutionary and are most closely related and have closest structural similarity [7]. LM-111 is successfully used* in vitro* in order to adhere isolated primary sperm stem cells [164].

*Bone Marrow Hematopoietic Stem Cells In Vivo and In Vitro*. Historically many early cell-based* in vitro* studies with hematopoietic stem cells are performed on Engelbreth-Holm-Swarm sarcoma-derived LM-111; however, it is not a relevant laminin isoform for this type of cells. LM-511 and LM-411 are expressed in bone marrow; LM-211 expression is weak and is restricted to a different niche, arterioles, while LM-111 is not expressed at all. *β*1, but not *β*2, laminins are present in adult bone marrow [165, 166].* In vitro* human CD34+ cells adhere strongly to LM-511/-521, but not to LM-111 or LM-211. LM-511/521 stimulate proliferation in human hematopoietic progenitor cells and enable robust adhesion for variety of hematopoietic lineages. Unlike LM-111, LM-511/-521 coating is strongly adhesive for multipotent hematopoietic FDCP-cells [166–168].

#### 4.1.9. Limited Ability of Certain Mammalian Cells to Secret Their Own Matrix Adhesion Molecules

Certain cell types, though requiring signals from laminins, can survive and even maintain and proliferate for long term in* in vitro* cell culture systems that lack the crucial laminins. It is possible since many cell types can provide specific adhesion ECM molecules, which they utilize afterwards. For instance, keratinocytes express LM-332, which promotes keratinocyte migration and stabilization during wound healing process. ESC express LM-511/521 [155], which is beneficial for their maintenance and proliferation. During* in vitro* culturing, however, part of the ECM molecules expressed by cultured cells may partially drift away from the cells location. Though ESC can generate LM-511/521, it is not sufficient to promote spreading to such extent as LM-511 or LM-521 is immobilized on culture plate surface.

Certain other cell types, on the contrary, vitally depend on laminin isoforms that they cannot express themselves. For instance, insulin-producing *β*-cells in pancreatic islets require contact with niche-specific laminin isoforms that need to be provided by a different type of cells, namely, vascular endothelial cells [157]. We hypothesize that it may be an important biological reason for the fact that *β*-cells do not migrate away from the basement membrane and therefore stay closely localized to the bloodstream and sense the glucose levels.

### 4.2. Integrins and Other Receptors

Many cell membrane receptors interact with ECM. Integrins are evolutionary old receptor family in metazoan. They are the major cell adhesion receptors for ECM proteins, and they are important for cell-cell interactions too. By now, there are 18 *α* and 8 *β* subunits known in vertebrates that assemble into 24 heterodimeric transmembrane proteins with different binding properties. They are expressed in tissue-specific and stage-specific manner in the course of organ development. Integrins can serve as mechanical links between ECM and the cell cytoskeleton and convey signals across cell membrane both inwards and outside. Integrins may enable co-signaling with other receptors. They are essential for development, tissue organization, and function of the living organisms. Integrin gene knockouts in mice lead to various phenotype aberrations such as embryonic and perinatal lethality, defects in angiogenesis, hemostasis, and leukocyte function.

Since integrins lack enzymatic activity, their signaling depends on the assembly of protein complexes on the cytosolic face of the cell membrane. Binding of integrins to ECM molecules causes activation (integrin conformational change) that allows recruitment of cytoplasmic proteins such as talins and kindlins onto the integrin intracellular domains. These protein complexes interact with the cytoskeleton and recruit protein kinases activating various signaling pathways. The signaling events can affect proliferation, differentiation, survival/anoikis, polarity, motility of cells, and gene expression in them. Conformational changes in integrins that increase affinity for ligands and integrin clustering that increases avidity for ligands are two major mechanisms of integrin activation. Notably, it has been shown that external force applied to the cell can strengthen integrin-receptor binding and activate integrins [169].

An additional level of complexity comes from the fact that integrins can cooperate with other cell receptors. Thus, Telci et al. [170] have shown that interaction of fibronectin with a cell surface receptor Syndecan-4 can restore adhesion of fibroblast even if direct interaction between fibronectin and the cells is blocked. This binding activates protein kinase C*α* (PKC*α*) that, in turn, interacts with *β*1 integrins. This restores cell adhesion and the associated actin stress fiber formation that is accompanied by activation of focal adhesion kinase and ERK1/2 mitogen-activated protein kinases. On endothelial cells, integrin *α*v*β*3 and VEGFR2 interact and are able to promote activation of each other [171]. This leads to signaling activation and, consequently, changing patterns of cell behavior. Therefore, integrin signaling is a complex and cell specific process.

Dystroglycan, sulfated glycolipids, Lutheran receptor, Syndecans, discoidin domain receptors, leukocyte-associated immunoglobulin-like receptor-1, and CD-44 [172] are examples of non-integrin cell membrane receptors that can interact with ECM molecules. Similar to integrins, the interactions can modulate cell behavior and convey signals across the cell membrane.

#### 4.2.1. Distinct ECM Molecules Engage Distinct Integrin Receptors in Stem Cell Cultures

As we mentioned previously, different natural ECM molecules were successfully implemented to allow adhesion and improve survival in ESC cultures* in vitro*. Though all of those enabled adhesion, the interaction involved different integrin receptors. For instance, interaction of ESC with vitronectin allowed self-renewal via contact with integrin receptor *α*V*β*5, adhesion of ESC to fibronectin occurs via integrin *α*5*β*1, and adhesion to laminin-111 occurs via integrin *α*6*β*1 [149]. Adhesion of human ESC to biologically relevant laminin-521 occurred via integrin *α*6*β* [153], as well as laminin-511 [152] and LM-E8 fragment, a truncated version of laminin-511 and laminin-521 [173].

#### 4.2.2. Matrix Epitopes Interaction with Cell Receptors: Not as Simple as Exclusive Affiliation

One might expect that the reason for laminin diversity and highly specific effect could be that every specific laminin isoform would specifically activate one specific integrin receptor and thus activate one specific downstream signaling pathway leading to a certain cell behavior pattern.

However, the experimental data implies that though interaction with certain laminins indeed activates specific behavior patterns, the mechanism is not as straightforward. Plantman et al. have cultured primary adult dorsal root ganglion neurons on four different laminin isoforms: LM-111, LM-211, LM-332, and LM-511. The integrin-binding G-domains of those laminin isoforms belonged to four different laminin *α* chains: *α*1, *α*2, *α*3, and *α*5 respectively, each having its own pattern of integrin receptor associations. The cultured neurons formed long neurites when cultured on LM-511 and LM-111, but not on LM-411 or LM-211. Notably, though neurons spread axons on LM-511 as well as on LM-111, they engage different integrin receptors [160]. LM-411 formed adhesion contacts via the same integrin receptor *α*6*β*1 as LM-511; however, it did not initiate neuritis formation. LM-211 formed adhesion contacts via the same integrin receptors *α*3*β*1 and *α*7*β*1 as LM-111; however, it also did not initiate neuritis formation [160].

In the ESC culture systems, as we discussed previously, laminin isoforms LM-111, LM-511, LM-521, and Matrigel, a gel containing LM-111 as active compound, all engage integrin *α*6*β*1, unlike vitronectin and fibronectin that engage different integrin receptors. However, the laminin isoforms exert distinct effects on the cultured cells [151–153, 174]. Matrigel, a gel containing LM-111 as major cell adhesion molecule, is efficient in supporting survival of dissociated human ESC only in presence of ROCK inhibitor, a molecule that exerts very strong influence on cell cytoskeleton, signaling pathways, and cell behavior (which is reviewed in Section 2 devoted to perception of ECM elasticity by cells) [174], while laminin-521 allows the human ESC to survive as single cell culture in absence of ROCK inhibitor [153].

### 4.3. Affinity of Matrix Epitopes Interactions with Cell Receptors

Every ECM cell adhesion molecule has a specific pattern of interactions with cell receptors. We shall use the example of laminin family to illustrate the distinct biological effects resulting from distinction in affinity and specificity of different ECM molecules' interactions with cell receptors.

#### 4.3.1. Laminin *α* Chain G-Domains Specifically Interact with Cell Receptors

Each laminin is a trimeric molecule, the *α* chain of which bears a G-domain that specifically interacts with a range of integrin receptors (Figure 3). As we have discussed above, interaction of specific laminin *α* chains with integrin receptors is not exclusive interaction. Each laminin isoform interacts with a variety of integrin receptors, although with different affinities [113]. Despite the fact that laminin *α* chains have overlapping integrin interaction patterns, every *α* chain has exclusive biological function. Knockout animal models and analysis of genetic pathologies in human patients clearly indicated that damage of every *α* chain leads to a specific, very distinct phenotype (reviewed in [7]). *α*1 laminins are essential for the earliest stages of embryonic development, inducing polarization of pluripotent cells of blastocyst inner cell mass and thus making them capable of differentiation into three germ layers: endoderm, mesoderm, and ectoderm. Lack of *α*1 laminin chain results in blastocyst-stage embryonic death. In adult organism, however, *α*1 laminins have rather restricted expression patterns. *α*2 laminins are essential for neural and muscular systems; mutations in LAMA2, a gene encoding *α*2 laminin chain, are known to cause severe muscular and neural disorders. *α*3 laminins are specific for epithelial basement membranes and are essential for epithelial cells maintenance. *α*4 laminins knockouts did not exhibit such phenotype severity as other laminin *α* chains knockouts; however, lack of it in adult age causes a range of disorders resulting from by malfunction in vascular system, especially, microcirculation. *α*5 laminins are the most ubiquitous in adult organism and play important part during embryonic development. Lack of *α*5 laminins causes late-stage embryonic lethality in mice and cannot be compensated by other isoforms.

#### 4.3.2. Lessons from* In Vitro* Cultures of Embryonic Stem Cells

Long-term* in vitro* cultures of mouse ESC on *α*1, *α*3, *α*4, and *α*5 laminins undergo four distinct behavioral scenarios, despite the fact that they have been exposed to the same culture media and passaged to the same low seeding density. LM-111 cultured cells change morphology within 2 weeks only and acquire quiescence (long-term survival and stability in absence of proliferation). LM-411 cultured cells detach and die within several days. Cells cultured on LM-332 and LM-511, however, show similar morphology, extent of spreading and adhesion, and marker expression patterns. Those two cultures proliferate for over 150 doublings while maintaining the same high proliferation rate and consistently express pluripotency markers: Oct4, Sox2, and Nanog. However, drastic difference between those two cultures has been revealed by the true functional test, ability to give rise to whole functional mouse organism, being injected into inner cell mass of blastocyst. LM-332 cultured cell population has been shown to give rise to sick chimeric animals with low extent of chimerism which fail to undergo germline transfer; however, the LM-511 cultured ESC give rise to animals with strong chimerism [151], which in turn give rise to germline transfer generation (Domogatskaya, Rodin, and Tryggvason: unpublished manuscript).

### 4.4. Natural Processing of Affinity Domains

Certain ECM molecules undergo proteolytic processing of the domains responsible for binding the cell receptors. Such processing can (1) alter affinity and specificity of the ECM molecule interaction with cell receptors, (2) activate or suppress different signaling pathways, and therefore (3) change patterns of cell behavior.

#### 4.4.1. Proteolytic Processing of ECM Molecules Affinity Domains Changes Affinity and Specificity of Cell Receptors Interactions

Affinity domains, such as G-domains of laminin *α* chains, can be proteolytically processed during the course of natural maturation, thus changing pattern of cell receptors interactions and modulating cell behavior. G-domains of laminin *α* chains consist of five globular LG-modules (each domain named LG1 to LG5, resp.) and may be enzymatically processed, so that G-domain truncates from LG1–LG5 to LG1–LG3 and LG4-LG5 domains are removed. Integrin receptors are known to bind LG1–LG3 domains, while Dystroglycan and Syndecans are known to bind LG4-LG5 modules of unprocessed laminin G-domain. For example, it has been shown that shift from LG4-LG5 of unprocessed G-domain of epithelial LM-332 binding via integrin *α*3*β*1 to LG1–LG3 via integrin *α*6*β*4 changes epithelial cell behavior from migration to stable anchoring [175].

#### 4.4.2. Processed and Unprocessed ECM Isoforms: Effect on Pluripotent Cells* In Vivo*

As we discussed previously, LM-111 plays important role in very early steps of blastocyst inner cell mass cells differentiation. A knockout mouse model has been generated, wherein LM-111 is expressed in mature (processed) form only, in order to investigate the role of LG4-LG5 domain and biological function of the unprocessed LM-111 form. Knockout phenotype in mouse model, wherein the *α*1 laminin chain lacked LG4-LG5 terminal part, has early developmental disorders inconsistent with embryo survival [176].

#### 4.4.3. Status of Affinity Domains (Processed versus Unprocessed)

Level of affinity domain processing in matrix molecules is often a non-defined parameter in commercial preparations of ECM molecules. Moreover, even if an unprocessed isoform of ECM molecule is introduced to a cell culture, it may partially get processed by the proteolytic enzymes secreted by the cultured cells.

### 4.5. Role of Structural Non-Affinity Domains

#### 4.5.1. Full-Size Natural ECM Molecules versus Adhesive Peptides

Role of the affinity domains that interact directly with the cell receptors with certain affinity and specificity is certainly of high importance. Within the affinity domains certain epitopes, like peptides RGD or IKVAV, serve the same function of binding the receptors with high affinity and selectivity. Discovery of such peptides and/or domains gave rise to generation of artificial scaffolds wherein the adhesive epitope would be reduced to a specific peptide. Peptide-based materials have a number of practical advantages. First, cost production of peptides and small protein domains is low, which would make such scaffolds affordable to wide community. It is especially important for 3D matrices, which require far larger amounts of epitopes, compared to monolayer-coated 2D surfaces. Second, small size of the peptides allows achieving incredibly high local concentrations of the epitopes and thus induce multiple and strong focal adhesions. Silva et al. [131] claims that artificial fibrils bearing the IKVAV-peptide in concentrations exceeding the natural concentration of laminin-111 epitopes about 1000-fold allowed achieving significant effects on neural progenitor cells* in vitro*. Concentration epitopes of natural laminin-111 coating reach only 7.5 × 10^11^ epitopes/cm^2^, while use of 2D gel of peptide-composed nanofibers allows about 1000-fold higher epitope concentration of 7.1 × 10^14^ epitopes/cm^2^ [131].

However, there must have been evolutionary reasons for natural ECM molecules such as laminin isoforms, collagen isoforms, or fibronectin to evolve as very complex and large molecules even in simplest organisms like* Hydra*. Mutation in only one domain (not necessarily the adhesive one) of only one chain can cause malfunction of the whole molecule and result in severe disease or lethality of the whole organism. Evolution created laminins and collagens even in primitive organisms such as* Hydra* and made them essential for regenerative processes. The number of laminin chains and isoforms increased in order to enable the growing animal organisms:* C. elegans* and* Drosophila* fruit fly have already 2 laminin isoforms and 4 indispensable chains, following “evolutionary explosion” in Deuterostomia phylum. Molecular complexity, large size, and heterotrimeric form was maintained as new isoforms of laminins with new function emerged during evolution [7]. This knowledge leads to assumption that there may be biological reasons for molecular complexity, large size, and existence of domains that do not directly participate in cell adhesions.

Malinda et al. compared activity of 405 synthetic peptides from laminin-111 with the effects of the full length molecule in endothelial cell adhesion, migration, angiogenesis assay, and the rat aortic ring sprouting assay [177]. The effects of the peptides appear to be assay- and concentration-specific. A large number of active sites on laminin-111 molecule have been identified. Some of the active sites have appeared to be endothelial cell type-specific. These results suggest that small synthetic peptides cannot be a fully functional replacement of large ECM molecules.

#### 4.5.2. Implications for Stem Cell Research

Laminins interact with cells primarily via C-terminal G-domains of their *α* chains. These domains contain protein motifs that are responsible for binding to various integrins, Dystroglycan, sulfated glycolipids, Lutheran receptor, and some more. Nevertheless, other parts of laminin molecules can significantly affect interaction with cells. Miyazaki et al. [173] showed that E8 fragment of LN-511 supports proliferation of dissociated human pluripotent stem cells (PSCs). The whole LN-511 molecule enables self-renewal of human pluripotent stem cells (PSCs) passaged in cellular clumps [152] but lacks an ability to support survival of human pluripotent stem cells (PSCs) dissociated into single cell suspensions [153]. The other laminin isoform, LN-521, facilitates survival of dissociated human pluripotent stem cells (PSCs) even at a slightly higher degree than the E8 fragment of LN-511 [178] suggesting that laminin *β*1 chain negatively influences the survival of the cells.

#### 4.5.3. Proteolytically Degraded ECM Molecules Preparations

Preparations of ECM molecules used in research are often the proteolytically degraded, truncated forms of the natural molecules. Unlike the processing of laminin G-domains, which is a natural maturation process, such processing part of artificial technological process allows extracting the ECM molecules from highly cross-linked and tightly packed tissue scaffolds. Such proteolytic treatment may alter the functional properties of the extracted ECM molecules. For example, commercial forms of collagens I, II, and III may be atelocollagens or protocollagens that differ significantly in functional characteristics.

Wondimu et al. [179] have demonstrated that certain commercial batches of tissue-extracted laminins were lacking some functional domains. It is important to consider that certain batches of ECM molecules may be a heterogeneous mixture of molecules truncated to different extent and thus sending mixed messages to the cultured cells.

### 4.6. One Complex Molecule as Dual Signaling Hub

There is evidence that some of the adhesion ECM molecules may function as molecular machines with dual receptor binding capacity in one molecule.

#### 4.6.1. Fibronectin: Two Distinct Active Peptides Specific for the Same Receptor Type

An amino acid sequence Pro-His-Ser-Arg-Asn (PHSRN) is situated in fibronectin close to RGD site and has been proved to bind to the same integrin receptors as RGD. These peptides, RGD and PHSRN, spaced by 4 nm, result in an increase in markers of osteoblastic cell function [127]. PHSRN has been proved to enhance the spreading of cells that are attached to substrates containing the RGD peptide [180]. Recent study that the display of RGD and PHSRN induce the osteoblastic differentiation of MSCs when combined with the nanogroove topography but without any osteogenic supplements has been also found [181].

#### 4.6.2. Laminins: Distinction between *β*1 and *β*2 Isoforms

*α*5 laminin isoforms, such as LM-511, LM-521, and recombinant fragment LM-E8, allow supporting self-renewal (proliferation) of human ESC, but LM-521 has been proven to be superior compared to LM-511 [152, 153, 173]. The difference between the two isoforms is *β*1 versus *β*2 chain. It is probable that the *β*2 laminin chain, unlike *β*1, has additional cell signaling properties. For instance, clear difference has been discovered between *β*2 and *β*1 laminin isoforms in neural signaling. Despite high structural similarity, *β*2 and *β*1 laminins sometimes act as antagonists, and *β*1-isoforms cannot replace lack of *β*2-isoforms in *β*2-knockout models that suffer severe neural disorders. Nishimune et al. have demonstrated that *β*2 laminins, apart from interacting with cell receptors via *α* chain G-domain, can also bind and cluster together voltage-gated calcium channels within synaptic cleft, thus recruiting other presynaptic components, and thus enable neurotransmitter release from motor nerve terminals [20].

### 4.7. Cooperation between Extracellular Matrix Signaling Systems and Growth Factor Signaling Systems

#### 4.7.1. Growth Factor Accumulation by ECM: Spatial Presentation of the Captured Growth Factors to the Cell Growth Factor Receptors by ECM Molecules

It was long noticed that ECM molecules can accumulate growth factors and thus act as depot for long-term storage of growth factor and controlled release of those. It appears that ECM may not merely store the growth factors but also may present them to the growth factor receptors on cell surface [19].

#### 4.7.2. Concept of Co-Signaling: Cell Interaction with Relevant ECM Molecules Makes the Adherent Cell Permissive for Growth Factor Stimulation

Signaling pathways triggered by different receptors, for instance, by cell membrane ECM receptors and growth factor receptors (GFRs), can collaborate with each other in a form of co-signaling (see Figure 2 for illustration). Co-signaling sometimes leads to synergetic effects that neither of the participating activated cell membrane receptors can cause alone. Although it is not required, different cell receptors often physically colocalize at the cell membranes. There are several kinds of co-signaling such as independent regulation of the same pathway, enabling of growth factor dependent GFR signaling by ECM receptors, quenching of growth factor dependent GFR signaling by ECM receptors, direct activation GFRs in the absence of the growth factors by activated ECM receptors, and amplification of GFR signaling by activated ECM receptors [182]. Thus, cooperation of signaling associated with LN-111 binding to integrin receptors and signaling associated with prolactin binding to its receptor constitutively activates signal transducer and activator of transcription protein 5 (STAT5) in mammary epithelial cell cultures and sustains mammary-specific gene expression [121]. Signaling triggered by neither LN-111 nor prolactin alone is able to sustain mammary-specific function of the cells. Stenzel et al. have demonstrated that only cooperative signaling of LN-411 through *β*1-containing integrins and VEGR-A signaling through its receptor VEGFR2 are sufficient to induce physiologically functional levels of Dll4 expression and regulate vascular density* in vivo* by inducing Dll4/Notch signaling pathway [156]. In mice, absence of the laminin signal leads to reduced Dll4 expression and pathological vessel branching in the retina.

Signaling via ECM receptors can also collaborate with cell membrane receptors that are involved in cell-cell interactions. Individualized human ESC die from anoikis [183], which can be prevented by cooperation of integrin-mediated ECM signaling and cadherin-mediated cell-cell signaling. We have shown that contact with laminin-521/E-cadherin substratum enables clonal survival of human ESC [153, 178]. Importantly, the cloning efficiency depends on weight/weight ratio of the components suggesting that the relative concentration or densities of the components affect the behavior of the cells. Neither LN-521 nor E-cadherin alone allows efficient survival of individualized human ESC. 

*There Are Other Types of Co-Signaling.* Co-signaling occurs not only between ECM signaling and growth factor signaling systems, but also between ECM and cell-cell-contacts signaling [153] or ECM and ECM (another type) signaling [170].

## 5. Synergy between Different Aspects of Cell Niches

We have discussed over 15 aspects of ECM, each of which in certain setup for a certain stem cell type can be influential for behavioral choice. Since stem cells are much more versatile in reactions to external stimuli, they must be especially sensitive to changes in ECM, whether physical, spatial, or molecular. It is especially striking how highly precise is the decision-maker system for stem cells, when they are within their natural niches. Chances for random behavior are extremely low; otherwise, the stem cells either might have expanded without restrictions or would diminish or differentiate randomly into cell types that are not specific for the particular niche.

Stem cells within their natural niches* in vivo* sustain high functional stability through years. They retain ability to react properly to changes in surrounding niche, should it be a need for quiescence or proliferation, survival or apoptosis, or differentiation to a certain cell type.

However, stem cell community cultured* in vitro* develops into a mixture of heterogeneous cell types. The behavioral choices of specific cells within the same cultured stem cell population after months of culturing may be spontaneous and random rather than uniform and predictable. The reason for random behavioral choices may be that cells receive contradicting messages from their artificial milieu. In contrast, within the natural niches all the surrounding contacts are in developed concordance with each other to support one behavioral pattern, but not a challenge of contradicting pathways.

The cues that cells receive from ECM should be in concert with messages delivered by soluble factors (growth factors and hormones) and contacts with neighboring cells also. Following are several examples of synergy between ECM, growth factors, and cell-cell contacts.

### 5.1. Co-Signaling

It is a concept based on synergy of two cell receptors interacting with two different ligands. In Section 4, we discussed examples of co-signaling of ECM-binding receptors with (1) growth factor receptors, (2) cell-cell adhesion contacts, and (3) another ECM molecule-binding receptor.

### 5.2. Specific Epitope Signaling Requires Proper Polarity to Render Cells Sensitive to Growth Factor Stimuli

Contact with biologically relevant ECM molecules may be necessary but not sufficient in order to make cells sensitive to growth factors stimuli by the co-signaling mechanism. Xu et al. [121] have demonstrated that contact with relevant matrix molecule (LM-111) and stimulation with relevant growth factor (prolactin) alone are not sufficient to enable function in mammary epithelial cells (*β*-casein expression that is naturally driven by stimulation by prolactin). The cells grown in traditional adhesive culture fail to respond to prolactin stimulation, since the polarity is not relevant and the prolactin receptor becomes spatially segregated from its natural ligand, prolactin. However, once proper polarity has been restored by culturing the cells as LM-111 microincapsulated 3D aggregates, prolactin stimulation initiated the STAT5 activation pathway and resulted in stimuli-driven *β*-casein expression.

Cell polarity is important when designing a culture system for a specific cell type. It would be different for each specific cell type; thus, for mammary epithelial cells and insulin producing pancreatic *β*-cells it would be relevant to introduce growth factors from the basal side, while for vascular endothelial cells it would be relevant to introduce soluble factors present in bloodstream from the apical side. Conventional 2D adhesive culture system allows bringing soluble factors from the apical side, while one can use more advanced systems such as Transwell plates, 3D gels, or microencapsulated 3D cell clusters in order to introduce the soluble factors from the basal side.

### 5.3. One Complex Molecule Bearing Two Different Epitopes: A Dual Signaling Hub

We have previously discussed ability of laminins to serve as dual signaling hubs. For instance, *β*2 laminins are essential for signal transmission in neural system and, in case of *β*2 laminin knockout, cannot be replaced by similar *β*1 laminins [184, 185]. The reason for such difference had not been obvious, since laminin *α* chains, but not *β* chains, are known to interact with integrin and other cell receptors. Work of Nishimune et al. [20] has provided insights into why the *β*2 chain, being part of a laminin trimer, would be so indispensable.

There is evidence that other ECM molecules, such as fibronectin, can also function as dual signaling hubs. Synergy ligands on fibronectin are also shown to alter the affinity of adjacent adhesion epitopes to cell receptors. As described above, RGD and PHSRN sequences are situated close to each other on fibronectin molecule and bind the same type of integrin receptor. They have been proved to affect synergistically the spreading and differentiation of the cells, including MSCs [127, 180, 181].

### 5.4. Co-Signaling and Growth Factor Presentation

Aside from the concept of co-signaling, regarding role of ECM ligands in making cells perceptive to growth factors stimuli [182], ECM can also serve a function of capturing the soluble growth factors and even presenting them to the growth factor receptors [19]. Notably, those are two different types of ECM molecules: the ones adhering the cell and making it perceptive (like laminins) and the ones adhering the growth factors and presenting them to the cells [186].

### 5.5. Molecular Structure Defines Supramolecular Assembly and, Therefore, Physical Properties and Epitope Localization

Laminin-111, the first laminin species isolated, can polymerize into 3D gels in the presence of calcium ions at room temperature [187]. High extent of crosslinking, which has strong influence on physical properties and 3D structure, is due to the fact that short arms of *α*1, *β*1, and *γ*1 chains comprising LM-111 all can associate with one another with high affinity [187], except for association *β*1-*β*1.

It has been later discovered, however, that not all laminins *α*, *β*, and *γ* chains can interact with each other [188]. Moreover, truncated *α*3A and *α*4 laminins lack the *α* chain short arm and, therefore, cannot form stable 3D structures in homogenous solution. Epithelial laminin 3A32, after proteolytic processing, which is part of the molecule maturation, acquires a rod-like shape lacking *β* and *γ* short arms, and it is not capable of self-assembly.

Since basement membranes* in vivo* are often composed of not just one but several laminin isoforms, the proportion between those allows achieving the supramolecular organization with a proper extent of cross-linking. That determines the physical and epitope localization characteristics best serving the basement membrane function.

### 5.6. Synergy of Stiffness and Epitopes

Signals from ECM epitopes modulate stem cell commitment, governed by matrix stiffness. More pronounced these modulations are seen on stiffer gels, because cell spreading and contraction of stiffer substrates depend more on strength of cell adhesions, which in turn are based on ECM epitopes. In one of the first studies of stiffness-epitope interplay, Rowlands et al. have investigated the myogenic and osteogenic potential of various polyacrylamide (PAA) gel substrates that are coated with covalently bound tissue-specific ECM proteins (collagen I, collagen IV, laminin, or fibronectin). Osteogenic differentiation has been found to occur significantly only on collagen I-coated gels with stiffness 80 kPa. Myogenic differentiation occurs on all gel-protein combinations that have stiffness levels exceeding 9 kPa with peak expression of myogenic marker seen on gels with a modulus of 25 kPa coated by fibronectin [22]. In more recent study, immobilization of RGD has been shown to promote proliferation and differentiation of MSCs, especially for the case of the stiffer gels [87]. It is worth pointing out that sensation of matrix stiffness by cells does not depend on strength of attachment of epitope-bearing molecules to the matrix scaffold (protein tethering): it has been shown by Wen et al. [49] that the surface density of collagen fibers covalent anchoring points has no impact on how cells deform the underlying substrate.

### 5.7. Synergy of Stiffness and Topography

Studies of interplay between stiffness and topography focus on thresholds in which stiffness or topography alone plays a major role, yet practically excluding synergy of these clues. To date, it is established that on small adhesive areas, comparable to cell size, the size of adhesive island is the most significant physical signal for cell differentiation, compared to stiffness [48]. At larger adhesive areas matrix elasticity is becoming more significant clue for MSC [48]. Results of the study by Li et al., in which the interplay of the three factors has been investigated, suggest that the stiffness is predominant to size and shape of the topographical features (pillars or grooves), in regulating osteogenic differentiation of rat bone marrow stem cells (BMSC) [54]. Stiffness is also predominant factor in regulating proliferation of these cells, but in this case size of topographical features appears to dominate over the shape [54]. Nevertheless, more studies are needed to reveal interplay between stiffness and topography clues for stem cells.

## 6. Future Perspectives

Many important aspects of the role of ECM in cell differentiation have been elucidated in the last decade. This new knowledge results in many technological breakthroughs, especially in the area of regenerative medicine. However, experimental studies often focus on certain specific aspects of ECM-cell interaction neglecting other important parameters.

This review addresses the issue of synergy between various aspects of this quite complicated system of dynamically interacting components. That synergy exists between the cues the cell receives from matrix, neighboring cells, and growth factors. In Section 5 devoted to synergy we present several illustrations to the idea that a biologically relevant element, taken alone and out of the niche context, may not be sufficient to provide strong positive effect in absence of certain other biologically relevant factors. However, combination of a number of relevant elements will give strong positive effect the same as it happens in native tissues.

High-throughput screening arrays, such as [189] or [190], that allow evaluating many thousands of different compounds in cell assays are successfully used to identify the promising candidate molecules for cell culture systems [191–193]. However, if a high-throughput screening experiment involves variation of only one niche parameter (whether artificial scaffold or growth factor), while ignoring other critical parameters, it may not be highly effective for certain cell types that depend on a specific combination of several niche factors.

**Figure 4:**
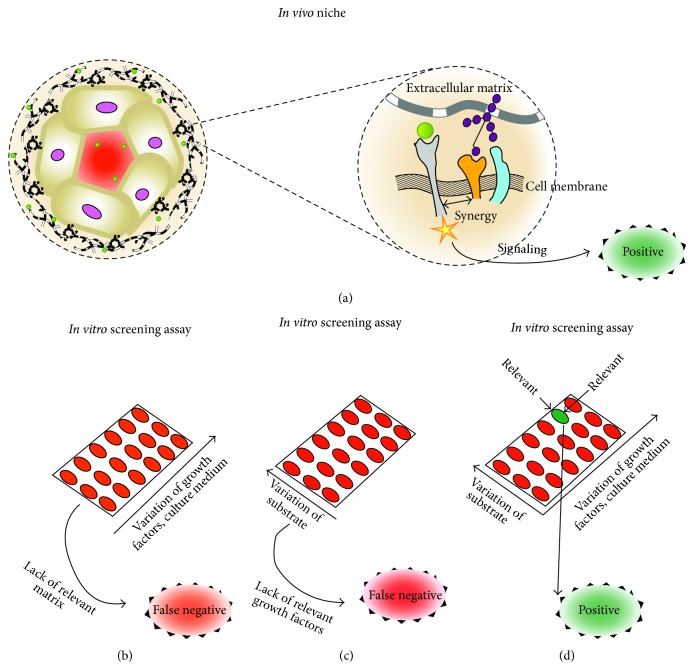
*Biological relevance as a key to developing functional in vitro culture systems*. There is a need for developing advanced, highly functional, robust, and long-term lasting* in vitro* culture systems for cells and organoids. In order to identify biologically active scaffolds, adhesion epitopes, and growth factors, high-throughput array approach is often used. It allows unbiased screening of large libraries of soluble compounds (proteins, peptides, and inorganic substances), as well as libraries of matrix scaffolds. However, sometimes biologically active compounds show false negative result, if the other aspects of the system are not biologically relevant. (a) The natural niche of a specific cell type can often serve a prototype for developing a highly functional* in vitro* culture system. Synergy of biologically relevant extracellular matrix cues and growth factors may be required in order to enable well-regulated cell function. (b) When a library of growth factors is analyzed in cell-based high-throughput screening array, the highly biologically active compounds may be identified as “false negatives” if the adhesive scaffold is not biologically relevant and does not enable co-signaling. (c) Also, if a library of scaffolds, whether natural, artificial, or mixed, is analyzed in cell-based high-throughput screening array, the truly functional scaffold may fail to provide the desired effect, if the cell culture medium lacks biologically relevant soluble factors that take part in co-signaling. (d) It is advisable, therefore, to arrange the high-throughput screening assays that would screen for a combination of growth factors library versus a scaffold library. The positive hit that can be missed in single-library screening may be identified in double-library cross-screening array.

It would hardly be possible to screen all the combinations of all the important niche aspects (in our review we list over 15 such aspects, and the libraries of certain aspects, such as growth factors or scaffold materials, sometimes include as much as 10,000 compounds). We suggest that biological relevance of the niche factors could be a helpful guidance for choice of such niche aspects as polarity, biologically relevant adhesion molecule, or stiffness to implement in* in vitro *screening setup (see Figure 4). As niche features are specific for each certain cell type, one should carefully examine the particular* in vivo* niche (combining the knockout animal studies, histological analysis, and mechanoelastic and viscoelastic evaluations) to identify the relevant values for the critical parameters. Notably, the niche features revealed for one cell type may not be applicable to other cell types.

## 7. Conclusions

1. Over 15 properties of ECM affect stem cell behavior and fate. Physical properties include stiffness (or elasticity); viscoelasticity; pore size and porosity; amplitude of static and dynamic deformations of the matrix (tensile, compressive, or shear); and frequency of cyclic deformations. Spatial properties include dimensionality (2D or 3D) of the scaffold; cell polarity; thickness of the substrate layer underlying the cell; surface area and geometry of adhesion surface; microscale topography of the surface; epitope concentration; epitope clustering characteristics (number of epitopes per cluster, spacing between epitopes within cluster, spacing between separate clusters, cluster patterns, and order or disorder in epitope arrangement); and size, shape, and level of disorder of nanotopographical features. Biochemical properties include diversity and structural complexity of matrix molecules, affinity and specificity of epitope interaction with cell receptors, role of non-affinity domains, ability to assemble into complex supramolecular structures due to structural domains of specific shape, and co-signaling by several epitopes and/or growth factors.
2. Synergy between different niche cues from ECM, including physical, spatial, and biochemical, as well as soluble factors and cell-cell contacts, in many cases is essential to provide long-term robust cell function.
3. Biological relevance of all the niche aspects, including physical, spatial, and biochemical, may be an effective approach in order to design functional* in vitro *stem cell culture systems.

## Figures and Tables

**Table 1:** Summary of elastic moduli^*∗*^ of mammalian tissues.

Tissue type	Range of stiffness	Testing method	Reference
Bone marrow	<0.3 kPa	Atomic force microscopy	[41]

Brain	0.5–1 kPa	Indentation	[33, 34]

Endothelial basement membrane	2-3 kPa	Atomic force microscopy	[28]

Hypodermis (skin layer)	2 kPa	Millimeter indentation	[29]

Adipose tissue	2–6 kPa	Indentation	[11, 30]

Liver	2–6 kPa	Ultrasound elastography	[44]

Fibrosis (liver)	8–12 kPa	Ultrasound elastography	[44]

Media layer of arterial wall	2–15 kPa	Atomic force microscopy	[61]

Muscle tissue (relaxed)	8–20 kPa	Atomic force microscopy	[11, 43]

Heart tissue (at diastole)	10–20 kPa	Atomic force microscopy	[46]

Chondron (matrix around chondrocytes)	20–30 kPa	Micropipette aspiration	[37]

Precalcified bone (matrix around osteoblasts)	20–50 kPa	Atomic force microscopy	[10]

Fibrous tissue (heart)	20–60 kPa	Atomic force microscopy	[42]

Dermis (skin layer)	35 kPa	Millimeter indentation	[29]

Muscle (skin layer)	80 kPa	Millimeter indentation	[29]

Skin	100 kPa	Optical coherence elastography	[31]
	210 kPa	Millimeter indentation	[30]

Arterial wall	0.3–1 MPa	Tensile tests	[25]

Cartilage	450–800 kPa	Indentation	[38]
	20–50 kPa	Nanoindentation	[39]

Tendon	2–8 GPa	Tensile tests	[36]

Bone (cortical bone)	10–20 GPa	Ultrasonic and microtensile test	[35]

*Tissue culture plastic *(*TCP*)	1–10 GPa		[50, 56]

^*∗*^Presented are the values, which have been measured at the lowest strain and lowest strain rate to approximate the elastic behavior of the tissues at rest, that is, at small deformations. The values of Young's modulus for any given tissue measured using different methods of deformation typically span several orders of magnitude [32]. Results for indentation are typically lower than for tensile tests. For unambiguity, testing methods are mentioned. Mechanical testing methods are summarized in Table 2.

**Table 2:** Methods to assess physical characteristics of matrices and tissues, used in stem cell studies.

Method/device	Description	Application variants and aspects	Studies using this method
Young's modulus and complex shear modulus assessment			
Note: different methods can give different results for the same tissue			

*Macromicroscale *(whole tissue specimen elasticity is measured)			
Micromechanical testing systems	Measuring applied stress and strain during deformation of the specimen of millimeter size (compression, tension, or indentation test); to measure viscoelastic properties (storage and loss modulus) *dynamic mechanical analysis* is used; that is, sinusoidal stress of certain frequency is applied and the strain in the material is measured	Compression test Tensile test Indentation	[53, 55, 132] [61, 78] [56, 58]
			
Indentation method	In indentation experiments, a rigid indenter (e.g., a plane ended cylinder, a cone shaped tip, or a sphere) is pressed against the tissue and shear moduli are calculated from the applied load and extent of tissue deflection	Nano- and macroscopic indentation	[33]
			
Rheometer	Two parallel plates or two coaxial cylinders with a narrow gap in-between are moving relative to each other, imposing shear stress on material squeezed in the gap; displacement and force are measured; thus, shear storage modulus and loss modulus are calculated and usually used to measure viscosity or rheology of fluids	Liquefaction stress of thixotropic gels Viscoelasticity measurement	[87] [12]
			
Magnetic resonance elastography	Shear waves inside the sample are induced by sonic mechanical vibrator on the surface of the sample; then, the shear wave propagation is recorded with a magnetic resonance technique and the image is assessed to generate a shear stiffness map	Non-invasive method for measuring stiffness in small samples	[76]
			
Ultrasound elastography	An external force is applied to the studied tissue and the resulting displacement and the generated strain are then mapped by ultrasound imaging; the external force can be static (compression, shear) and dynamic (shear waves propagation, whose speed is directly related to the medium shear modulus)	Non-invasive method for measuring tissues stiffness in patients, for instance, suffering from liver fibrosis	[44]

*Nanoscale *(local elasticity is measured)			
Atomic force microscopy (AFM)	The specimen is subjected to indentation by nanometer size indenter; force-indentation distance profiles are collected and analysed with a Hertz cone model to compute the elastic moduli	Nanoindentation	[10, 49, 52, 68, 89]
			
Micropipette aspiration	The tip of a small micropipette is brought in contact with a sample and a series of equal steps in pressure are applied; the length of the sample aspiration representing an equilibrium deformation is determined for each pressure Young's modulus is calculated from the experimental length–pressure data (using particular theoretical model)	Used for thin matrix samples to assess local characteristics of pericellular matrix	[37]

Pore size assessment			

Advanced microscopy methods	The porosity and pore sizes of dried hydrogels are examined by microscopic imaging	Scanning electron microscopy (SEM)	[49, 51]
		Confocal microscopy	[51]
			
DNA electrophoresis	The radius of gyration of extended DNA may be used to estimate the effective maximum pore size of the hydrogel		[49]
			
Pore size through permeability measurement	Measuring permeability of the gel, that is, fluid flow velocity through the gel under certain pressure, allows calculating mean pore size		[89]

**Table 3:** Experimental materials used to study the effect of matrix physical properties on stem cells *in vitro*.

Polymer substrates	+ advantages/− disadvantages	Examples	Method to vary elasticity	Reference
*Natural polymers *				
	+ good adhesion capabilities + mimicking of natural ECM	Collagen-I gel (1 wt.%) and collagen-hyaluronic acid mixture	By the concentration of water soluble carbodiimide (EDC) as a crosslinking agent to produce matrices of 1–10 kPa with pore sizes about 50 *μ*m	[49]
				
Collagen-based gels		Collagen-I gel (1 wt.%)	By thickness of solution before polymerization: 500 *µ*m for soft, few microns for stiff	[78]
				
	+ injectable, for brain repair + biodegradable	Gelatin-hydroxyphenylpropionic acid (Gtn-HPA) hydrogel	By chemical cross-linking by an enzyme-mediated oxidation reaction	[12, 22]
				
Hyaluronic acid (HA) gel	+ mimicking of natural ECM − pure HA does not permit cells to adhere; must be coated by cell-adhesion proteins predominantly elastic, with very low viscous component	Thiol modified HA (HA-S) gel coated with gelatin	By concentration of PEG-diacrylate as a cross linker	[50]
		Hyaluronic acid (HA) 1 wt.%	By the concentration of water soluble carbodiimide (EDC) as a crosslinking agent, pore size 80 *μ*m	[87]

*Synthetic polymers *				
Polyacrylamide (PAA) gel	pure PAA is fully elastic material but can be used to produce viscoelastic gel + plenty of methods to tune its stiffness without changing porosity or even pore size − pure PAA do not permit cells to adhere; must be coated by cell-adhesion protein	PAA gel coated with type 1 collagen	By different ratios of acrylamide/bisacrylamide concentrations in a mixture	[55, 62]
		PAA matrices of varying pore sizes but the same stiffness	—//—	[59]
		PAA gels coated with covalently bound tissue-specific ECM proteins (collagen I, collagen IV, laminin, or fibronectin)	By different ratios of acrylamide to cross-linking agents bisacrylamide, ammonium persulfate, and tetramethylethylenediamine (TEMED)	[52, 57]
		Viscoelastic PAA gels in which the loss modulus is varied whilst the storage modulus is kept constant	—//—	[55]
				
Polyethylene glycol- (PEG-) based gels	+ high elasticity + cytocompatibility + ability to be photopolymerized	Polyethylene glycol-(PEG)	By percentage of PEG polymer in the precursor solution	[10]
		3D thixotropic polyethylene glycol-silica gel (turns liquid under certain stress)	By weight percentage of fumed silica particles incorporated into the gel	[54]
		Polyethylene glycol diacrylate (PEGDA)	By altering the duration of the photopolymerization process	[68]
		Mixture of PEG-dimethacrylate (PEGDMA) and PEG-methacrylate	By different ratios of PEG-dimethacrylate (PEGDMA) and PEG-methacrylate in a mixture	[51, 132]
				
Polyvinyl alcohol (PVA)	+ stiffness gradient formation	Polyvinyl alcohol (PVA) hydrogel produced by the hydrolysis of polyvinyl acetate	By gradual freezing	[53]
				
Poly dimethylsiloxane (PDMS)	+ biocompatibility viscoelastic material used for micropatterned islands formation	Polydimethylsiloxane (PDMS)	By mixing base and cross-linker at different ratios by using temperature gradient to create a gradient in the crosslinking density of siloxane	[56, 58, 58, 64, 89]
				
Electrosrun fibers: micro- or nanofibres drawn from a polymer solution using an electric charge	+ ECM-mimicking nanofiber structure, allowing control over fiber diameter + allowing achieving high porosities or large pores + ability to be photopolymerized + useful to form 3D matrix + useful to create substrates of different topology	Polyethylene glycol dimethacrylate PEGdma and poly(ethylene oxide) (PEO)	By adjusting the photopolymerization time	[61]
		Poly(*ε*-caprolactone) (PCL)	By different ratios of solvents	[65]
		Biodegradable polyurethane (PU) synthesized with using poly(*ε*-caprolactone) (PCL) as the soft segment	By different ratios of solvents (2,2,2-trifluoroethanol and N,N-dimethylacetamide)	[60]

**Table 4:** Summary of MSC differentiation dependence on substrate stiffness.

Lineage	Matrix stiffness, *E*	Material	Testing method	Reference
Neurogenic	0.1–1 kPa	2D polyacrylamide gels, collagen coated	Nanoindentation using atomic force microscopy	[10]
	~1 kPa	2D polyvinyl alcohol hydrogel	Compression test	[53]
	1 kPa	3D type I collagen gel and hyaluronic acid gel	Compression test	[55]
	~1-2 kPa	2D gelatin-hydroxyphenylpropionic acid gel	Dynamic shear deformation 1%, 1 Hz using rheometer	[57]
	6.1 kPa	2D polyacrylamide gel (PAA)	Compression test	[54]

Gliogenic	10 kPa	3D type I collagen gel and hyaluronic acid gel	Compression test	[55]

Vascular endothelial cells	2-3 kPa	3D polyethylene glycol dimethacrylate (PEGdma) nanofiber hydrogel	Tensile test	[61]

	2.5–5 kPa	3D alginates-agarose hydrogel with RGD	Compression test	[132]
Adipogenic	4 kPa	2D polyacrylamide gel (PAA)	Nanoindentation using atomic force microscopy	[49]
	1.5 kPa, 6 kPa	2D polydimethylsiloxane (PDMS)	Tensile and macroscopic indentation tests	[56]

Myogenic	7–17 kPa	2D polyacrylamide gels (PAA), collagen coated	Nanoindentation using atomic force microscopy	[10]
	12–15 kPa	3D polyethylene glycol dimethacrylate (PEGdma) nanofiber hydrogel	Tensile test	[61]
	~30 kPa	2D gelatin- hydroxyphenylpropionic acid gel	Dynamic shear deformation 1%, 1 Hz using rheometer	[57]
	>9 kPa: 25 kPa, 80 kPa	2D polyacrylamide gel, coated with collagen, fibronectin	Dynamic mechanical analysis	[22]

Cardiomyocytes	45 and 65 kPa	3D thermosensitive hydrogel (PAA and HEMA-PTMC)	Tensile test	[46]

Osteogenic	11–30 kPa	3D alginates-agarose hydrogel with RGD	Compression test	[132]
	15–100 kPa	2D polydimethylsiloxane (PDMS)	Tensile and macroscopic indentation tests	[56]
	24 kPa	2D polyvinyl alcohol hydrogel	Compression test	[53]
	25–40 kPa	2D polyacrylamide gels, collagen coated	Nanoindentation using atomic force microscopy	[10]
	30 kPa	2D polyacrylamide gel (PAA)	Nanoindentation using atomic force microscopy	[49]
	42 kPa	2D polyacrylamide gel (PAA) collagen coated	Tensile test	[78]
	46.7 kPa	2D polyacrylamide gel (PAA)	Compression test	[54]
	~60 kPa	2D gelatin–hydroxyphenylpropionic acid	Dynamic shear deformation 1%, 1 Hz using rheometer	[59]
	80 kPa	2D polyacrylamide gel coated with collagen, fibronectin	Dynamic mechanical analysis	[22]
	190 kPa–3.1 MPa	2D polydimethylsiloxane (PDMS)	Nanoindentation	[58]
